# Annealing Effects on Cu Migration in the Colloidal Synthesis of Pd-Chalcogenides Nanoheterostructures

**DOI:** 10.1021/acs.nanolett.5c02469

**Published:** 2025-07-31

**Authors:** Suvodeep Sen, Niraj Nitish Patil, Ankita Bora, Manoj Palabathuni, Temilade Esther Adegoke, Kevin M. Ryan, Kevin Rossi, Shalini Singh

**Affiliations:** † Department of Chemical Sciences and Bernal Institute, 8808 University of Limerick, V94 T9PX Limerick, Ireland; ‡ Department of Materials Science and Engineering, Delft University of Technology, 2628 CD Delft, The Netherlands

**Keywords:** Nanoheterostructure, Colloidal Synthesis, Cation Migration, Metal Chalcogenide

## Abstract

Heterostructuring nanocrystals into a modular metal–semiconductor configuration enables tunable and novel functionalities. Such combinations at the nanoscale equip hybrid structures with unique electronic, optical, and catalytic properties unobserved in single-phase materials. Here, we report the hot-injection synthesis of Pd-Cu_3_Pd_13_S_6.65_Te_0.35_ nanoheterostructures (NHCs) from PdCu nanoalloy seeds. First, the growth of Pd-rich chalcogenide nanocrystals was initiated over the preformed PdCu surface through simultaneous sulfidation and tellurization, followed by their transformation into Pd-Cu_3_Pd_13_S_6.65_Te_0.35_ NHCs. By strategically employing moderate-temperature annealing, we achieved the complete migration of Cu^+^ due to the higher reactivity of Cu in comparison to Pd at that temperature, establishing a novel mechanistic relationship between cation mobility and temperature. This strategy enables controlled semiconductor domain formation and targeted metal migration. The NHCs showed efficient and stable electrocatalytic hydrogen evolution with low Tafel values in acidic media, outperforming conventional nanoelectrocatalysts. Computational analysis identified the active sites responsible for the observed catalytic performance.

## Graphical abstract

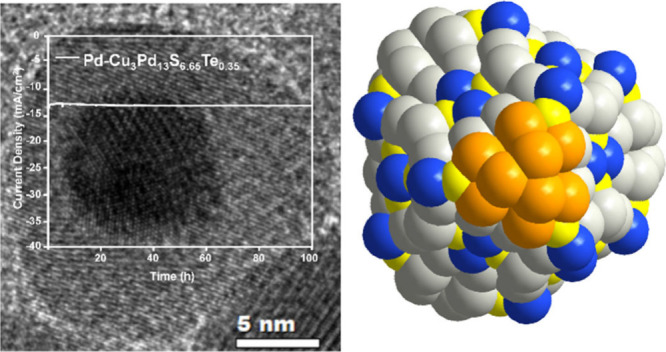

The increasing demand for materials with tailored properties has driven the need for greater precision in controlling the dimensionality and uniformity to customize their properties for specific applications.^1–7^ Inorganic nanoheterostructures (NHCs), designed by combining different materials, create synergistic interactions that enhance chemical,^8–10^ optical,^11,12^ and catalytic properties^13,14^ of advanced solid-state systems. Integrating metal and semiconductor segments into a single NHC presents significant synthetic challenges. To form cohesive hybrid nanocrystals (NCs), the synthesis must suppress homogeneous nucleation, cation exchange, and metal diffusion,^14,15^ achievable through precise control of concentration,^16,17^ ligand engineering,^18,19^ and temperature.^4,20^ Selective deposition on target seeds can be directed by optimizing reaction conditions that suppress cross-nucleation or parasitic monomer condensation in the reaction media.^2,14,21,22^ For instance, when two chemically analogous metals, X and Y, are heated to a temperature that activates atomic diffusion, atoms migrate across or along the interface, forming a homogeneous XY alloy. During subsequent chemical treatment at moderate temperatures, this interface dynamically shifts due to the differing diffusion rates (or ion mobilities) of X and Y. Such an interplay of diffusion kinetics between different metal ions has led to the formation of NHCs with intriguing functionalities.^23–26^ However, the inherent kinetic and thermodynamic constraints have limited the exploration of bimetallic heterodimer/alloy seeds chemicalisation compared to monometallic systems.^3,27–31^ This lack of control has impeded the integrated design and synthesis of such NHCs, limiting their adoption in next-generation energy technologies. Again, rising energy demand underscores the need for sustainable nanomaterials with superior performance. In this context, strategic material and synthesis design is key to uncovering bimetallic alloy chemicalisation mechanisms and producing NCs for energy applications. Notably, catalytic approaches like electrocatalysis are pivotal for efficient energy conversion, emphasizing the role of advanced nanomaterial design in future energy solutions.^32^ Developing such a dual-purpose electrocatalyst is both timely and impactful. Therefore, innovative synthetic strategies rooted in partial chemicalisation within the conventional seeded-growth framework are essential to overcome these challenges.^33–38^

In this letter, by leveraging the temperature-dependent cation migration to control heterostructuring, we demonstrate the synthesis of Pd-Cu_3_Pd_13_S_6.65_Te_0.35_ NHCs from PdCu heteroatom seeds using a colloidal hot-injection method. The inherent catalytic activity of Pd and Cu made them compelling choices as active metal centers. Meanwhile, S and Te were chosen over the more conventional S–Se pair due to the substantial ionic size disparity between S^2–^ and Te^2–^, enabling investigation of its impact on morphology and catalytic performance. This approach involved mobility-influenced cation migration reactions on presynthesized NC seeds, offering significant synthetic control for programmable structural diversification. A schematic trajectory of this reaction mechanism is depicted in Scheme 1. The atomic models were designed using fast Fourier transform (FFT) from electron microscopy images with Diamond Crystal Impact software, based on crystallographic information files 01-086-4068 and 04-017-1480. When presynthesized PdCu seeds were subjected to simultaneous sulfidation and tellurization in the presence of oleylamine (OLAm) and 1-octadecene (1-ODE) as reducing agents and solvents, the kinetically favorable PdCu-Pd_16_S_7–y_Te_*y*_ NHCs were first formed as intermediates. Owing to copper’s faster (or higher) migrating ability, the intermediate eventually transformed into thermodynamically stable Pd-Cu_3_Pd_13_S_6.65_Te_0.35_ NHCs as the final product. The synthesis was performed at different anion injection and annealing temperatures to elucidate the underlying reaction mechanism while keeping the precursor ratios unchanged. At moderate temperatures (180 °C), the differences in cation mobility enabled Cu cations to diffuse and redistribute throughout the Pd_16_S_7–y_Te_*y*_ semiconductor while Pd remained less reactive and suppressed. However, at higher temperatures (200 °C and above), the competing migration of both cations promoted cross-nucleation, resulting in the formation of PdTe and Cu_3_Pd_13_S_6.65_Te_0.35_. A detailed description of the synthesis protocol and purification is provided in the Supporting Information (SI). The favorable formation of PdTe at high temperatures is guided by the lower enthalpy of formation of PdTe over PdS.^39^ Furthermore, the compositional and morphological advantages were examined for electrochemical hydrogen evolution reactions (HER). The Pd-Cu_3_Pd_13_S_6.65_Te_0.35_ NHCs exhibited exceptional HER activity, achieving a low overpotential (η) in acidic media and remarkable long-term stability, maintaining a steady chronoamperometric response for over 100 h. In the quest to rationalize the catalytic performance of the NHCs, a thorough investigation of the hydrogen adsorption energy was conducted employing advanced density functional theory (DFT) calculations. The interplay between adsorption modes, site coordination environments, and hydrogen adsorption energies is thoroughly examined and discussed in a subsequent section.

**Scheme 1:**
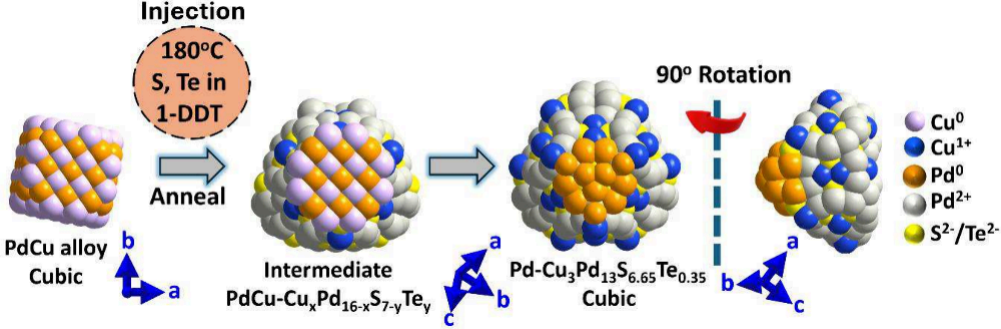
Representation of Proposed Overall Reaction Pathway for Synthesis of Pd-Cu_3_Pd_13_S_7‑x_Te_*x*_ NHC

The powder X-ray diffraction (XRD) of the different samples synthesized via injecting chalcogen precursors into presynthesized PdCu alloy NCs at different temperatures (experimental details provided in the Supporting Information) is carried out and presented in Figure 1a. The initially synthesized PdCu alloy exhibited a cubic phase (Figure S1); however, on introducing the anionic precursors {S + Te + 1-dodecanethiol (1-DDT)} into the reaction mixture first resulted in the formation of Pd_16_S_7‑y_Te_*y*_ over PdCu at 180 °C (cornsilk). The XRD pattern of the time-dependent aliquot collected 1 min after the injection of (S + Te + 1-DDT) revealed that the (111) and (002) planes of PdCu alloys were predominantly present at that stage, exhibiting the most intense peaks (Figure S2). Meanwhile, peaks corresponding to the semiconductor phase had just begun to emerge. This was followed by the subsequent migration of Cu cation that catalyzed the transformation into a heterostructure with the composition Pd*-*Cu_3_Pd_13_S_6.65_Te_0.35_. The XRD spectrum (Figure 1a) confirms NHC formation with cubic Pd nanoparticles integrated with cubic Cu_3_Pd_13_S_6_._65_Te_0_._35_ after annealing at 180 °C for 15 min. However, when annealing was carried out at elevated temperatures (200, 220, and 230 °C), cross-nucleation of cubic Cu_3_Pd_13_S_6.65_Te_0.35_ and hexagonal PdTe NCs was triggered, as can be seen in Figure 1a (these phases are shown in lavender). In contrast, the XRD intensity of cubic Pd gradually diminished, signaling the complete incorporation of Pd into the reaction at higher temperatures. This is confirmed by the XRD heat map of different samples at 2 theta values of ∼31° and ∼39° (Figure 1b). Under thermodynamic control, surface tension governs the wetting of secondary materials, which is influenced by the surface structure and lattice compatibility of the semiconductor, here Cu_3_Pd_13_S_6.65_Te_0.35_ and PdCu alloy at heterojunctions. As such, complete wetting occurs only on selected PdCu seed facets, or partial wetting occurs on all surfaces due to significant interfacial lattice mismatch. This leads to discontinuous deposition of the secondary component. As a result, the as-synthesized NCs exhibit heterodimer configurations, where disk-like domains of the secondary material are attached to the seed substrate through small-area bonding heterojunctions.^11,34,40–42^ This selective control facilitated the formation of Pd-Cu_3_Pd_13_S_6.65_Te_0.35_ NHCs resembling those derived from monometallic NC seeds. To unravel the mechanisms governing phase and stoichiometry in the colloidal Pd-Cu_3_Pd_13_S_6.65_Te_0.35_ NHCs, aliquots of the reaction mixture were sampled at different times throughout the annealing process at 180 °C (Figure S3). Aliquot XRD revealed NHC formation after 3 min of annealing, with extended annealing enhancing growth and crystallization. This thermal control influences whether one or both types of cations can fully mobilize and integrate during the secondary growth stage. Such directed migration is pivotal for initiating/avoiding cross-nucleation, where more than one material nucleates directly from the seed, impacting structural evolution and composition of NHCs. This determines whether a heterostructure forms or phase-separated NC systems emerge, as shown by the LaMer plot in Figure 1c. Ostwald’s step rule for nucleation dictates that a metastable phase emerges first, preceding its transformation into the thermodynamically stable state.^43^ Accordingly, in our system, PdCu-Pd_16_S_6.65_Te_0.35_ is likely to first form as the metastable phase.^44,45^ However, due to the fast kinetics of the reaction, aliquots collected after 1 min of reaction indicated the presence of PdCu-Cu_*x*_Pd_16‑x_S_7‑y_Te_*y*_ (confirmed by XRD in Figure S2). The slower reactivity of Pd at 180 °C facilitates the migration of Cu^1+^ into the Pd_16_S_7‑y_Te_*y*_ segment, followed by reorganization into stable Pd-Cu_3_Pd_13_S_6.65_Te_0.35_ NHCs.

**Figure 1:**
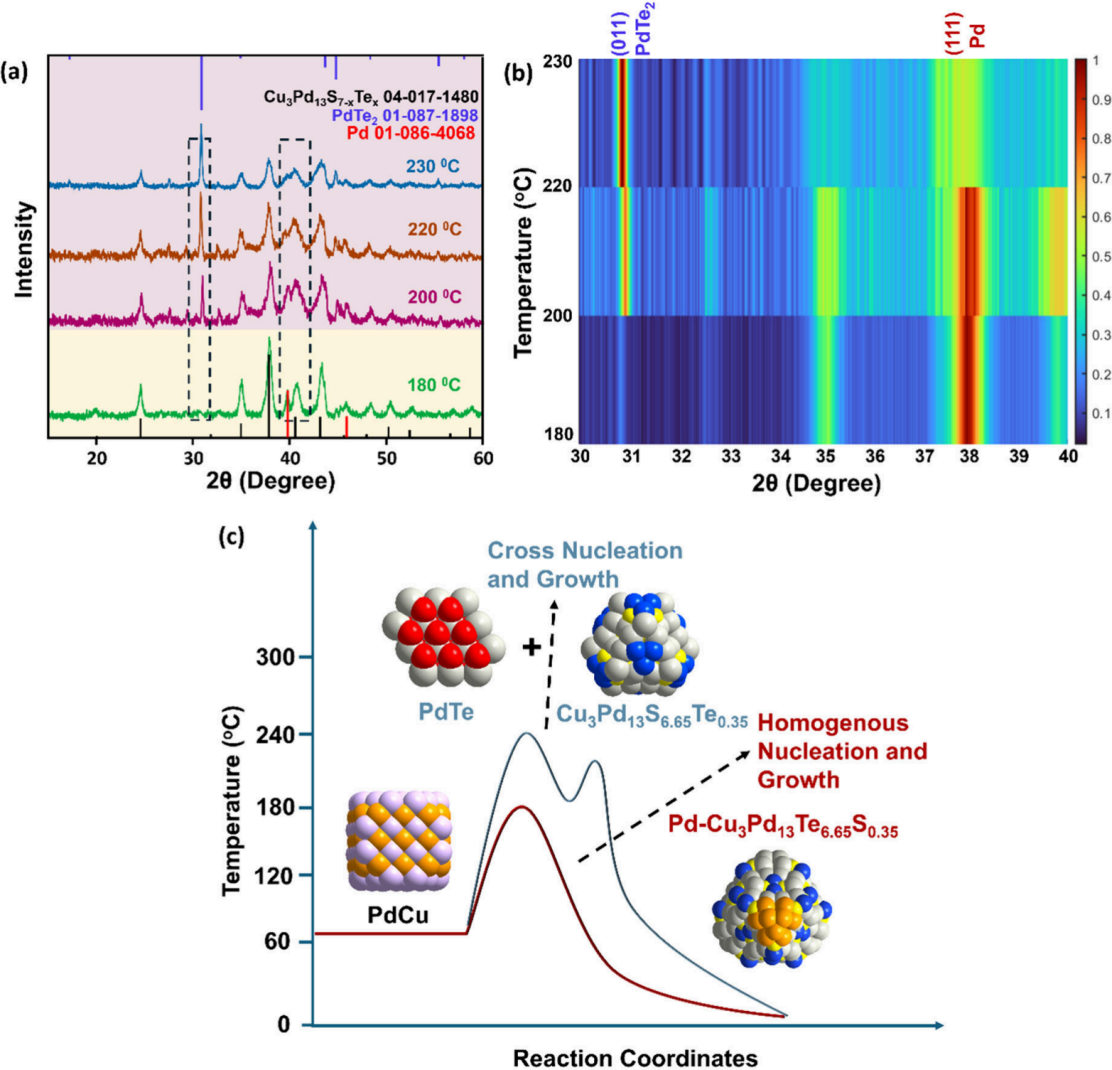
(a) Powder XRD patterns of Pd-Cu_3_Pd_13_S_6.65_Te_0.35_ NHCs from samples carried out at different reaction temperatures. Two regions in the XRD for NHC formation and cross-nucleation in (a) are highlighted with different colors (cornsilk and lavender). (b) Corresponding XRD intensity heat map from 2 theta values of 30 to 40°. The heat map was performed using MATLAB software. (c) LaMer plot representing the formation of Pd-Cu_3_Pd_13_S_6.65_Te_0.35_ NHCs vs (PdTe + Cu_3_Pd_13_S_6.65_Te_0.35_) NCs governed by precursor reactivity trends across different reaction temperatures. The gray, orange, purple, blue, red, and yellow spheres denote Pd, Pd^2+^, Cu, Cu^1+^, Te, and S, respectively.

Figure 2a and b present the transmission electron microscopic (TEM) and high-resolution TEM (HRTEM) images of Pd-Cu_3_Pd_13_S_6.65_Te_0.35_ NHCs synthesized at 180 °C. The obtained NHCs showed a nearly uniform size distribution, as shown in the low-magnification, inverted TEM image in Figure 2a. Additional TEM and HRTEM images of PdCu alloy and Pd-Cu_3_Pd_13_S_6.65_Te_0.35_ NHCs are presented in Figures S4 and S5, with size distribution histograms in Figure S6 (Supporting Information). Figure 2b presents the HRTEM images of NHCs and the heterojunction. The corresponding selected area FFT pattern of the heterojunction is shown in Figure 2c. The FFT pattern revealed the presence of a Pd lattice with the *d*-spacing value of ∼2.3Å corresponding to the (11–1) planes of the cubic Pd phase (*Fm*3̅*m*) when viewed along [011]. Again, while viewing along [001] direction, the *d*-spacing values of 2.3 Å corresponding to (400) planes of cubic Cu_3_Pd_13_S_6.65_Te_0.35_ (*I*4̅3*m*) were observed. This indicates that the (11–1) plane of Pd and the (400) plane of Cu_3_Pd_13_S_6.65_Te_0.35_ have the same *d*-spacing value, suggesting reduced lattice mismatch. But this matching of *d*-spacing was not observed in all NHCs; also, Pd appears to be partially embedded in the semiconductor domains, indicating variability in local structural alignment across the NHCs. An additional HRTEM image of the heterostructure, captured along a different crystallographic orientation, was also included in the analysis to provide complementary structural insights (Figure S7).

**Figure 2:**
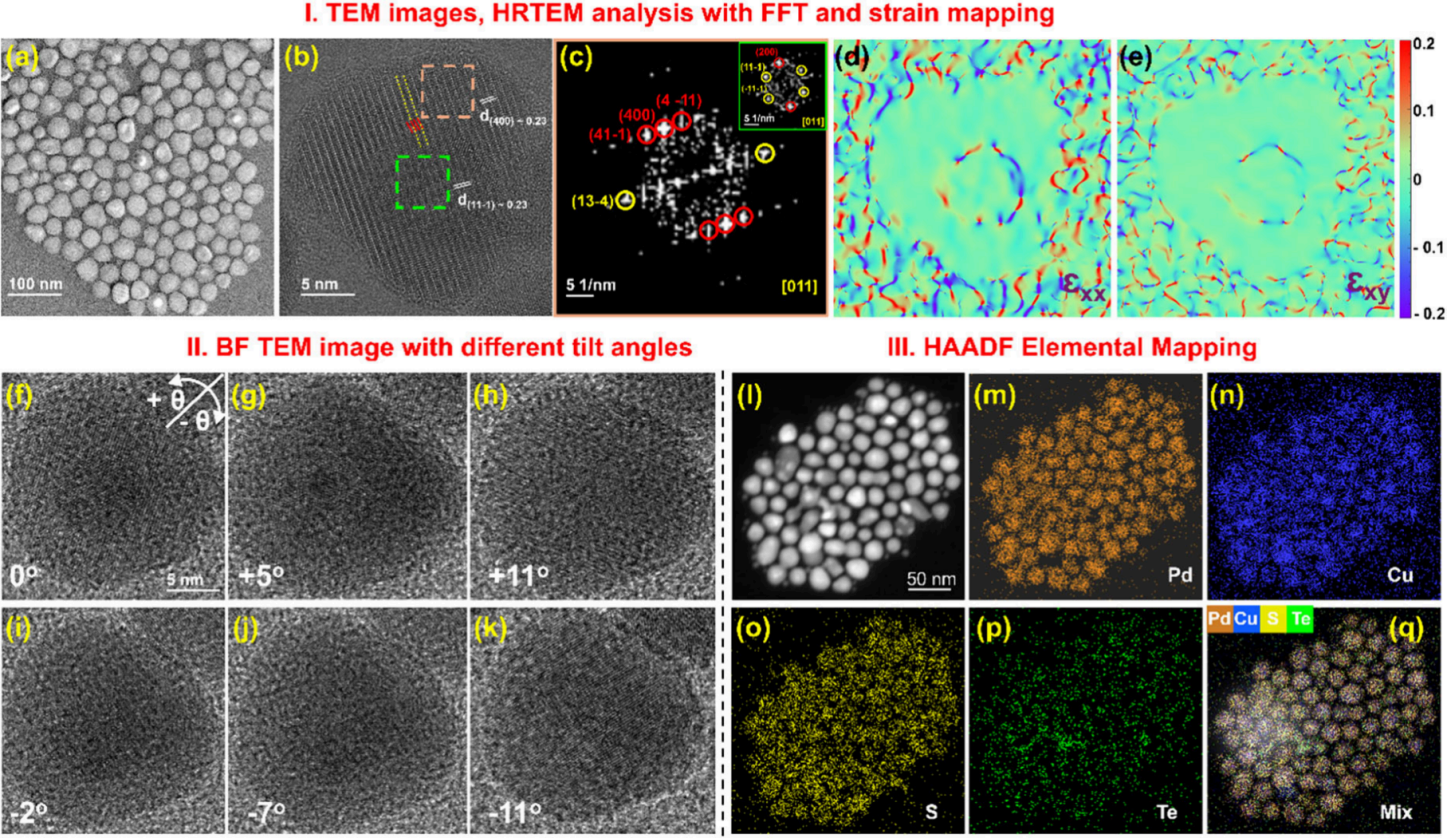
Panel I presents the electron micrographic images of Pd-Cu_3_Pd_13_S_6.65_Te_0.35_ NHCs in different resolutions: (a) Inverted TEM image of the NHCs. (b) HRTEM showcasing the heterojunction with the existence of strain and (c) the corresponding selected area FFT patterns of Cu_3_Pd_13_S_0.65_Te_0.35_ disks and Pd NPs (inset), respectively. (d, e) ε_*xx*_ and ε_*xy*_ (shear) strain maps show the presence of strain along the Pd/Cu_3_Pd_13_S_6.65_Te_0.35_ interface. The color gradient from blue to red represents strain values ranging from −0.1% to 0.1%, with green indicating zero strain. Areas near the NHCs lacking lattice fringes introduce noise into the data. Strain maps were performed using Strain++ software. Panel II depicts the BF-TEM images of tilting performed in (f–h) positive direction at θ = 0°, +5°, +11°, and in (i–k) negative direction at θ = −2°, −7°, −11°. Panel III presents (l) the HAADF-STEM image with an overlaid elemental map (m–q) of the synthesized NHCs.

Strain at the NHC interface is evident in Figure 2b (and other NHCs, SI), highlighted by yellow lines. To quantify this, geometric phase analysis was performed on the HRTEM image in Figure 2b. The resulting strain field component maps for ε_*xx*_, ε_*xy*_ are presented in Figure 2d and e. These maps exhibit a distinct contrast arising from tensile and compressive strain at the interface, underscoring the significant role of strain in governing NHC growth. This often happens when one domain is strained to match the lattice of the other domain. Strain maps for two additional HRTEM images are provided in Figure S8, further highlighting the strain at the interface. To resolve overlapping structures and confirm that the as-synthesized NHCs are Janus-type rather than core–shell, TEM tilting experiments were conducted, as presented in panel II, Figure 2f–k. The bright field (BF) TEM image in Figure 2f reveals one distinct, high contrast region almost at the center of the nanodisk at a 0° tilt angle. As the Bragg condition is met at varying locations within the crystal, the high-contrast region shifts and gradually fades, as seen in Figure 2f–k. To estimate the angles of crystal curvature from the zone-axis pattern image, a TEM tilt series was performed in bright field (BF) mode by tilting an individual NHC within the range of −11° < θ < +11°. At a 0° tilt (Figure 2f), the darker section of the Pd nanoparticle is distinct and nearly centered. When the NHC is tilted to +5.0° (Figure 2g), the darker Pd section shifts away from the center, with movement perpendicular to the tilt axis. As the tilt angle increases to +11.0° (Figure 2h), the Pd region gradually rotates outward from the center and eventually disappears. Notably, tilting in the negative direction (−2° to −11°) confirmed the same structural evolution, reinforcing the consistency of these observations and supporting the Janus-type nature of the structure. (Figure 2i–k). A complete tilt series ranging from −11° to +11° in low resolution on a collection of NHCs is presented in Figure S9. Furthermore, in panel III, Figure 2l, the high-angle annular dark field (HAADF) image of the NHCs presents the distinct regions of Pd nanoparticle in Cu_3_Pd_13_S_6.65_Te_0.35_, clearly showing a Janus-like interface. Again, the scanning transmission electron microscopy–energy dispersive X-ray spectroscopy (STEM-EDS) elemental mapping with an overlay of the NHCs (Figure 2m–q) affirms the presence of a Cu, S, Te, and Pd-rich composition in the NHCs. The line-scan for the region in Figure 2l is presented in Figure S10, enabling elemental analysis at the atomic level.

The controlled reaction was conducted at an elevated temperature (250 °C) to rigorously assess whether such thermal conditions would induce any significant transformation in morphology or crystal structure; however, no significant differences were observed in the NCs formation under the two reaction conditions (230 and 250 °C injection temperature). TEM images of cross-nucleation products formed at 250 °C are displayed in Figure S11. Thus, the annealing temperature is a decisive factor in dictating the role of the PdCu seed during the secondary nucleation–growth process. Also, the TEM and HAADF-STEM mapping images of intermediates collected after 1 min of reaction at 180 °C are provided in Figures S12 and S13. Based on these images, the synthesized NHCs exhibit a Janus morphology rather than a core–shell structure. The elemental composition was validated through inductively coupled plasma optical emission spectroscopy (ICP-OES) analysis (Table S1). The ICP-OES analysis confirmed the NHC stoichiometry of Pd-Cu_3_Pd_13_S_6.65_Te_0.35_. The absorbance spectra of PdCu seeds and Pd-Cu_3_Pd_13_S_6.35_Te_0.35_ NHCs are presented in Figures S14 and S15. The PdCu NCs exhibited absorbance primarily in the UV region, whereas the NHC displayed broad absorbance across the UV–vis region. Additional control experiments were conducted by varying the ODE/OLAm ratios and increasing the amount of S precursor for the synthesis of NHCs. Variation in OLAm concentration led to noticeable changes in the particle size of the NHCs, as illustrated in Figure S16. In contrast, increasing the sulfur content resulted in the formation of tadpole-like nanoheterostructures, as shown in Figure S17.

Although XRD, TEM, ICP-OES, and EDX measurements were consistent, they primarily offer insights into the stoichiometric composition and structural analysis, which might not necessarily reflect the surface characteristics. Therefore, to obtain information specific to the surface and near-surface region and the electronic state of the elements, X-ray photoelectron spectroscopy (XPS) analysis (Figure 3 and Figure S18) was conducted, which confirmed the presence of crystal-bound Pd, Cu, S, and Te in the NCs. Upon deconvolution of the high-resolution XPS spectra, the Pd 3d spectrum revealed two main doublets. The lower binding energy doublet (3d_5/2_ at ∼336.1 eV and 3d_3/2_ at ∼341.7 eV) corresponds to metallic Pd^0^, while the higher binding energy (3d_5/2_ at ∼337.8 eV and 3d_3/2_ at ∼343.1 eV) is consistent with the presence of Pd^2+^ species, which we attribute to Pd bound to chalcogen atoms (Te or S). This differentiation supports the coexistence of both zerovalent and oxidized Pd species, aligning with the Janus-like coordination environment. The Cu 2p spectra in Figure 3b exhibit a doublet peak with 2p_3/2_ at ∼934.4 eV and 2p_1/2_ at 952.1 eV, corresponding exclusively to the Cu^+^ species, while the doublet peak with 2p_3/2_ at ∼932.4 eV and 2p_1/2_ at 954.9 eV indicates the presence of small amounts of Cu^2+^ due to partial aerial oxidation, and the peak position is consistent with Cu–S or Cu–Te coordination rather than metallic Cu^0^ (which appears at slightly lower binding energies). The satellite peak with 2p_3/2_ at ∼944 eV and 2p_1/2_ at ∼962.1 eV in Cu is attributed to the shakeup effect, which is a characteristic of Cu (II)-containing samples but unobserved in Cu (0) or Cu­(I) species.^46^ The sulfur 2p spectra in Figure 3c revealed two doublets upon peak fitting. The first doublet, at a binding energy of 160.5 and 161.7 eV, is characteristic of metal-bound sulfide (S^2–^). A minor doublet, with 2p_3/2_ at 163.1 eV and 2p_1/2_ at 164.3 eV, corresponds to residual thiolate bonds from the 1-DDT used during the reaction as a capping agent. The peak area ratios indicate that the concentration of surface-bound thiols is low, suggesting they are present only as a minor component in the sample. Again, in Figure 3d, the Te 3d spectra at 583.8 and 585.6 eV confirmed the presence of Te^2–^ and traces of TeO_2_, respectively. Furthermore, the distinct N 1s peak at 398 eV (Figure S18) can be attributed to the presence of chemisorbed nitrogen species associated with surface ligands OLAm.

**Figure 3:**
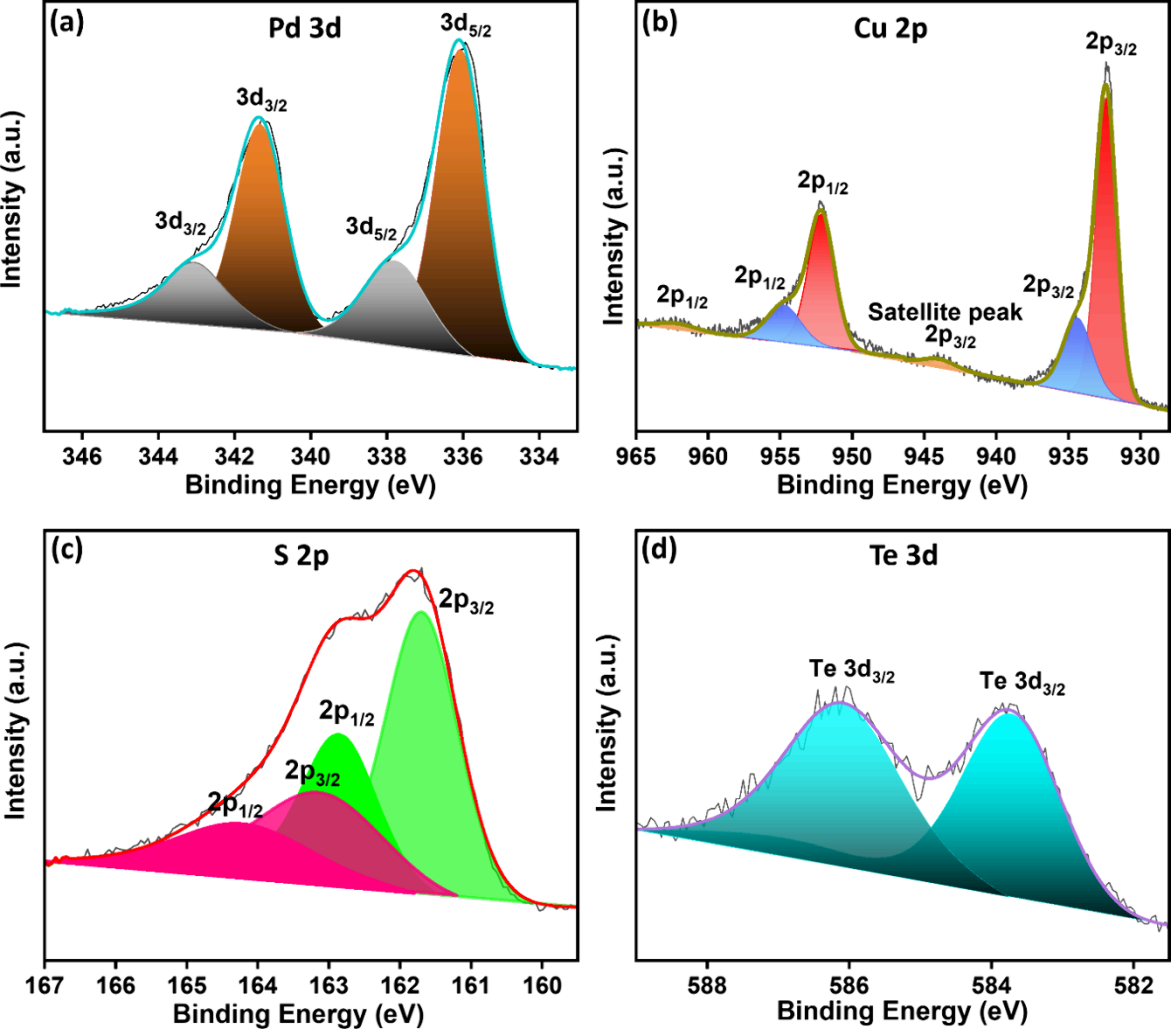
High-resolution XPS spectra of Pd-Cu_3_Pd_13_S_7‑x_Te_*x*_ NHCs for (a) Pd 3d, (b) Cu 2p, (c) S 2p, and (d) Te 3d.

The electrocatalytic performance of the as-obtained NHCs was evaluated using linear sweep voltammetry (LSV) polarization curves. A lower η and a higher current density (j) indicate superior electrocatalytic performance, reflecting enhanced efficiency and reaction kinetics. Figure 4a illustrates the HER activity of Pd-Cu_3_Pd_13_S_6.65_Te_0.35_ NHCs, carbon paper (CP), and commercially available state-of-the-art catalyst, Pt/C, in 0.5 M H_2_SO_4_. Pd-Cu_3_Pd_13_S_6.65_Te_0.35_ achieved a current density of 10 mA/cm^2^ at the lowest η value of 107 mV, demonstrating superior HER activity compared to previously reported metal chalcogenide-based electrocatalysts (Table S2). The enhanced HER activity of the Pd-Cu_3_Pd_13_S_6.65_Te_0.35_ NHCs is primarily attributed to the more electron-rich domains, which provide additional reductive sites for hydrogen ion reduction. This promotes efficient charge transfer between the metal and semiconductor, ultimately boosting the catalytic activity of the NHCs. The Tafel plots in Figure 4b for Pd-Cu_3_Pd_13_S_6.65_Te_0.35_ NHCs and Pt/C wire highlight the HER kinetics of the catalyst-loaded working electrodes. Their calculated Tafel slope values were found to be approximately 191 and 60 mV/dec for Pd-Cu_3_Pd_13_S_6.65_Te_0.35_ NHCs and Pt/C, respectively, suggesting improved mass and electron transport contributing to excellent HER kinetics.

**Figure 4:**
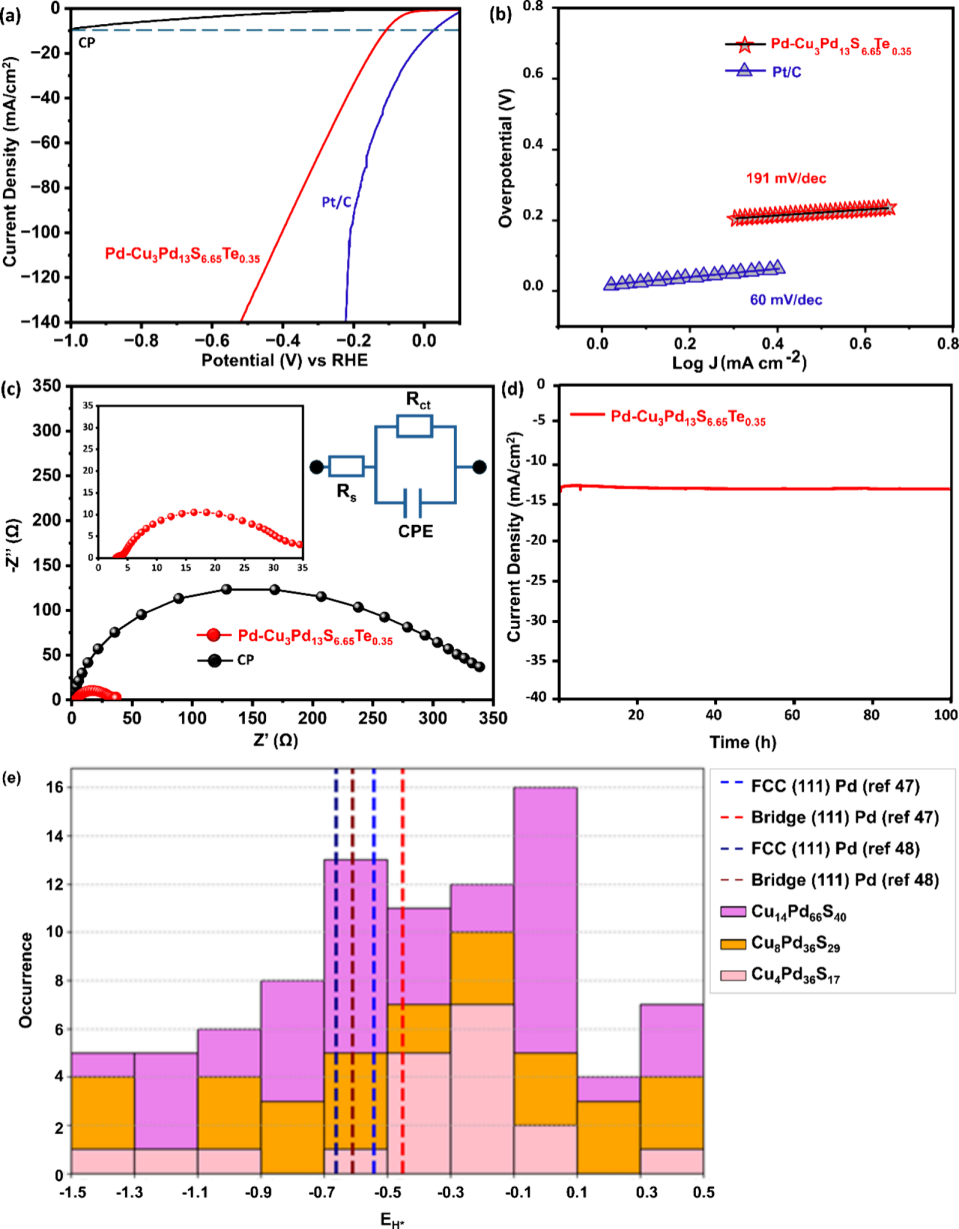
(a) Overlay of polarization curves of CP, Pd-Cu_3_Pd_13_S_6.65_Te_0.35_ NHCs, and Pt/C nanowires. (b) Plot of Tafel slope derived from potential (E) versus logarithmic current density (log J) from linear sweep voltammetry (LSV) of Pd-Cu_3_Pd_13_S_6.65_Te_0.35_. (c) Electrochemical impedance plot of carbon paper and Pd-Cu_3_Pd_13_S_6.65_Te_0.35_ at −0.35 V vs RHE, with an inset showing equivalent circuit model. (d) Chronoamperometric I–t measurements at −0.35 V vs RHE. All measurements were conducted in 0.5 M H_2_SO_4_ electrolyte. (e) Distribution of H* adsorption energies on nonequivalent adsorption sites present in model Cu_3_Pd_13_S_7–x_ nanocluster systems considered in this study. Reference H* adsorption energies for Pd (111) surface are also reported for ref 47. The heterogeneity in site distribution and the presence of sites with ideal H* adsorption energy parallel the experimental observation of high activity.

The electrochemical impedance spectroscopy (EIS) was conducted (Figure 4c), with Nyquist plots fitted to the inset circuit model, revealing charge transfer resistance (R_ct_) at the electrode–electrolyte interface. Table S3 (Supporting Information) summarizes that the Pd-Cu_3_Pd_13_S_6.65_Te_0.35_ NHCs exhibit a significantly lower R_ct_ value of 32 Ω, compared to 336 Ω for bare CP. The reduced resistance stems from strong interfacial interaction between Pd nanoparticles and Cu_3_Pd_13_S_6.65_Te_0.35_, boosting conductivity and shortening charge diffusion length. This enhances charge transfer and HER performance. The stability of the synthesized catalysts was investigated using chronoamperometric measurements at a constant potential of −0.35 V vs RHE (Figure 4d). The NHCs maintained a steady current density of approximately 14 mA/cm^2^ for 100 h, demonstrating excellent electrochemical stability. Additionally, postcatalytic characterization of the Pd-Cu_3_Pd_13_S_6.65_Te_0.35_ electrocatalysts via TEM, XRD, and XPS analyses is presented in SI (Figures S19–S21). Figure S19 presents the TEM images of postcatalytic NCs. The NCs appeared slightly agglomerated with minimal changes in their morphology. Similarly, Figure S20 presents the post-mortem XRD analysis, which shows that the crystal structure of the NC remained largely stable, although partial oxidation of Te to crystalline TeO_2_ was observed. In the XPS spectra (Figure S21), the Pd 3d spectra exhibited a pronounced decrease in the Pd^0^ component and a marked increase in Pd^2+^ species, indicating substantial Pd oxidation. The S 2p spectra showed only S^2–^ species, confirming successful exchange with short-chain inorganic ligands and demonstrating the chemical stability of S under electrochemical conditions. The Cu 2p XPS spectra remained stable after electrocatalysis, indicating preserved Cu chemical state, while significant oxidation of Te was observed. Minimal changes in crystal structure and surface composition confirm the catalyst’s structural robustness and long-term viability for hydrogen evolution. To rationalize the catalytic properties of Pd-Cu_3_Pd_13_S_6.65_Te_0.35_-like system, we assessed the Hydrogen Adsorption energy (E_{H*})-a descriptor of the activity of the system for HER-on Cu_3_Pd_13_S_7_ by DFT calculations (see SI, Section 3 for methodological details).^48^ Because Cu_3_Pd_13_S_7_ has a large unit cell, surface models including multiple replicas of the unit cell entail a (too) high computational cost. To efficiently screen E_{H*} on many inequivalent sites, we thus consider small-scale systems of different sizes (56, 91 atoms, and 120, Figure S22). These are obtained by relaxing spherical cuts of size 0.6–1 nm carved out from the Cu_3_Pd_13_S_7_ unit cell. In Figure 4e, we report the distribution of E_{H*} over the nonequivalent sites present in these systems. This preliminary exploration of the complexity in the H adsorption on Cu_3_Pd_13_S_7–x_-like system illustrates that there exists a small but non-negligible number of sites with an H adsorption energy resulting in high activity, e.g., comparable with the ones of hollow and bridge sites on a Pd (111) surface.^47^ An analysis of the relationship between adsorption mode (Top, Bridge, Hollow), adsorption site coordination, and H adsorption energy shows nontrivial relationships (Figures S23 and S24). We summarize that a delicate interplay between electronic and geometric (strain and coordination) all contributes to Cu_3_Pd_13_S_7_-like systems activity. Extensive systematic studies are needed to identify surface terminations with the highest activity and stability theoretically, and to guide the design and synthesis of NHCs for improved catalytic properties. In summary, DFT calculations on Cu_3_Pd_13_S_7_-like systems reveal a few highly active hydrogen adsorption sites, with E_{H*} values comparable to Pd (111) surfaces. The catalytic activity arises from a complex interplay of electronic structure, coordination, and strain, warranting further theoretical and experimental investigation.

In conclusion, we present the one-pot synthesis of Pd-Cu_3_Pd_13_S_6.65_Te_0.35_ NHCs by the seed-mediated method, where the annealing temperature plays a crucial role in governing cation migration and phase formation. This, in turn, dictates the extent of Pd incorporation into the heterostructure, thereby impacting their composition and shape. At moderate temperatures, selective Pd retention enables secondary nucleation, integrating Cu_3_Pd_13_S_6.65_Te_0.35_ with cubic Pd, while elevated temperatures induce cross-nucleation, forming hexagonal PdTe and cubic Cu_3_Pd_13_S_6.65_Te_0.35_. These NHCs exhibited good electrocatalytic performance and excellent stability in acidic media toward HER, characterized by a lower η value of 107 mV, reduced R_ct_ of 39 Ω, and consistent chronoamperometric response of 14 mV/cm^2^ for 100 h. Computational investigation of H adsorption on small Cu_3_Pd_13_S_7_-like systems showed that surface sites in these materials frequently display ideal adsorption properties for HER. Our findings highlight the crucial role of thermal control and material selection in heterostructuring, paving the way for discovering a range of novel NHCs with precisely engineered properties for a broad spectrum of applications, including electrocatalysis with excellent material stability.

## Supplementary Material



## Notes

Additional data of XRD, TEM, EDS, XPS, absorbance, analysis of the aliquot samples, control reactions, DFT, and experimental details (PDF)
