# Supplementary material for: Annealing Effects on Cu Migration in the Colloidal Synthesis of Pd-Chalcogenides Nanoheterostructures
Source: Nano Lett. 2025 Jul 31;25(32):12207–15. doi: 10.1021/acs.nanolett.5c02469 (PMC12356118; doi:10.1021/acs.nanolett.5c02469)
Supplement: Supplementary file 1 [file nl5c02469_si_001.pdf]

Annealing Effects on Cu Migration in the Colloidal Synthesis of Pd-Chalcogenides Nanoheterostructures

*Suvodeep Sen [1], Niraj Nitish Patil [1], Ankita Bora [1], Manoj Palabathuni [1], Temilade Esther Adegoke [1], Kevin M Ryan [1], Kevin Rossi [2], Shalini Singh [1]**

[1] Department of Chemical Sciences and Bernal Institute, University of Limerick, V94 T9PX Limerick, Ireland

[2] Department of Materials Science and Engineering, Delft University of Technology, 2628 CD, Delft, The Netherlands

Contents

1. Experimental 2
   1.1 Materials 2
   1.2 Preparation of Te-S-thiol stock solution 2
   1.3 Synthesis of Pd-$Cu_3Pd_{13}S_{6.65}Te_{0.35}$ NHCs 2
   1.4 Synthesis of PdTe and $Cu_3Pd_{13}S_{6.65}Te_{0.35}$ NCs 2
   1.5 NC Purification Procedure 2
   1.6 Preparation of Working Electrodes 2
   1.7 General Safety and Handling 3
2. Instrumentation 3
   2.1 Transmission Electron Microscopy 3
   2.2 X-ray Diffraction (XRD) Analysis 3
   2.3 X-ray photoelectron spectroscopy (XPS) 4
   2.4 Optical property measurement 4
   2.5 Electrochemical measurement 4
3. Computational Methods 5
4. Additional Characterisation 6
5. References 20

1. Experimental

1.1 Materials

Palladium acetyl acetonate ($Pd(acac)_2$, ≥99.95%), Copper chloride (CuCl, ≥99.95%), octadecene (ODE, tech. 90 %), oleylamine (OLAm, tech 70%), sulphur powder (99.98%), tellurium powder (Te-30 mesh, 99.997%), 1-dodecanethiol (1-DDT), and were purchased from Aldrich and used without any further purifications.

1.2 Preparation of Te-S-thiol stock solution

Inside the glovebox, Te powder (56 mg, 0.3 mmol) and S powder (6.4 mg, 0.1 mmol) were dispersed in 1 mL of 1-DDT in a 5 mL glass vial and then sonicated for 30 minutes to obtain a well-dispersed black solution.

1.3 Synthesis of Pd-$Cu_3Pd_{13}S_{6.65}Te_{0.35}$ NHCs

In a typical synthesis, 26 mg (0.1 mmol) CuCl and 44 mg (0.1 mmol) of $Pd(acac)_2$ were mixed with 3 mL of ODE and 2 mL of OLAm as a solvent and reducing agent, respectively, loaded in a 3-neck round-bottom flask. The reaction mixture is then evacuated at 120°C for 60 minutes (5 min ramp to 120°C). The temperature was then raised to 280°C under inert conditions (Ar flow), annealed for 20 mins (5 min ramp to 280°C), and then ice quenched. The flask temperature was again raised to 180°C, and the S-Te-thiol stock solution was swiftly injected into the reaction mixture. The solution was further annealed for 15 minutes at the same temperature. The entire process was repeated multiple times to ensure the reproducibility of the results.

1.4 Synthesis of PdTe and $Cu_3Pd_{13}S_{6.65}Te_{0.35}$ NCs

This synthesis process did not require a different reaction protocol; only the S-Te-thiol stock solution was injected and annealed at different temperatures (200, 220, and 230°C) that favoured the formation of PdTe and $Cu_3Pd_{13}S_{6.65}Te_{0.35}$.

1.5 NC Purification Procedure

The NCs, synthesized and mixed with 10 mL of ethanol, were poured into a 50 mL centrifuge tube and vortexed well. The dispersed NCs were centrifuged at 5000 rpm for 5 min. The pellet was collected and dispersed in 1 mL of toluene first, and 10 mL of IPA was added, sonicated, and vortexed to disperse the NCs nicely. The NC solution was again centrifuged at 5000 rpm for 5 min, and the process was repeated with toluene and acetone (1:9) and dried at 80 °C overnight in a vacuum.

1.6 Preparation of Working Electrodes

1 mg of the synthesized Pd-$Cu_3Pd_{13}S_{6.65}Te_{0.35}$ samples was dispersed in 200 µL of 5% isopropanol-Nafion solution (here, Nafion was used as a surface binder). The mixture was sonicated for 15 minutes to achieve a uniform dispersion, forming the catalyst ink. Finally, 30 µL of this catalytic ink was carefully drop-cast onto carbon paper (CP), which served as the

working electrode. The working electrode was dried at 70 °C for 1 hour to remove the volatile organic components.

1.7 General Safety and Handling

When handling chemicals for this experiment, strict adherence to safety guidelines is paramount, with safety data sheets (SDS) from the chemical supplier reviewed before use. Proficiency in regulating vacuum and maintaining an argon-filled inert atmosphere using the Schlenk line is crucial, as the experiment involves high-boiling-point solvents at elevated temperatures. Proper training in air-free synthesis and Schlenk line techniques is essential before beginning.

Personal protective equipment (PPE), including lab coats, gloves, and safety goggles, must always be worn. All chemicals should be handled or measured in a glovebox or fume hood according to their respective SDS. Special attention must be given to certain substances due to their hazardous nature. Oleylamine, for example, is highly corrosive and toxic, necessitating careful handling in the fume hood with appropriate PPE. 1-Dodecanethiol, known for its corrosiveness, skin irritant properties, and pungent odour, must also be handled exclusively inside a fume hood, with any spills promptly cleaned up.

During evacuation procedures, using a liquid nitrogen trap is critical to condense hazardous gases produced during the reaction. An argon flow should be maintained using a bubbler to regulate the system and prevent direct exposure to gaseous impurities.

2. Instrumentation

2.1 Transmission Electron Microscopy

For TEM analysis, the NCs were dispersed in hexane and drop-cast on continuous carbon-coated 300 mesh nickel grids. Low-resolution and high-resolution TEM (HRTEM) and dark-field scanning transmission electron microscopy (DFSTEM) were conducted by using 200 kV Talos F200i field-emission microscope equipped with a Gatan UltraScan CCD camera and EDAX Genesis energy dispersive X-ray spectroscopy (EDS) detector and Thermo-Fisher Scientific FEI double-aberration-corrected monochromatic Titan Themis with a double tilt holder was used for aberration-corrected TEM and scanning transmission electron microscopy analysis. For analyzing the HRTEM data, interplanar distances and particle orientation were determined from the selected area FFT analysis using GMS3 software.

2.2 X-ray Diffraction (XRD) Analysis

XRD analysis was performed on the nanocrystals (NCs) drop-cast films deposited on a flat surface of p-type boron-doped silicon with zero background. The measurements were carried out using a PANalytical Empyrean instrument equipped with a Cu Kα radiation source (λ = 1.5418 Å) and a 1-D X’celerator strip detector. The diffractometer operated at 40 kV and 40

mA. The powder XRD (PXRD) patterns of the resulting NCs were analyzed using standard JCPDS files available in PDF5 database.

2.3 X-ray photoelectron spectroscopy (XPS)

XPS analysis was conducted using a Kratos AXIS ULTRA spectrometer equipped with a monochromatic Al Kα (1486.58 eV) X-ray source. Calibration was carried out based on the C 1s peak at 284.8 eV. Data processing, including spectrum construction and peak fitting, was completed using CasaXPS software. The XPS measurements were performed on vacuum-dried nanostructure samples to ensure accuracy and consistency in the analysis.

The reaction mixture was then collected in a syringe and injected into a centrifuge tube containing hot ethanol (50°C). Nanocrystals were then precipitated by centrifugation and purified using an ethanol–acetone mixture as an anti-solvent. The final product was dispersed in hexane or chloroform for further characterization.

2.4 Optical property measurement

Optical absorbance spectra were recorded using a Cary 5000 UV–vis–NIR spectrophotometer. The samples, dispersed in toluene, were placed in quartz cuvettes with a 1 cm path length, and measurements were performed in double-beam transmission mode with a lamp changeover at 350 nm.

2.5 Electrochemical measurement

All EC measurements were conducted at room temperature using an electrochemical workstation with a standard three-electrode setup in a 0.5 M H_2SO_4 solution. The as-prepared Pd-$Cu_3Pd_{13}S_{6.65}Te_{0.35}$/CP composites served as the working electrodes, with an Ag/AgCl electrode as the reference and a Pt wire as the counter electrode. All measured potentials were converted to the reversible hydrogen electrode (RHE) using the equation E(RHE) = E(Ag/AgCl) + 0.059pH + 0.197. Linear sweep voltammetry (LSV) was performed within a potential range of 0 to -0.8 V vs RHE at a scan rate of 5 mV s^{-1} for the HER. Tafel plots were generated using the Tafel equation ($\eta = a + b \log j$), where j represents current density, b is the Tafel slope, and a is the intercept. Electrochemical impedance spectroscopy (EIS) measurements were taken across a frequency range of 100 kHz to 100 mHz, with an AC voltage amplitude of 10 mV. Catalyst durability was assessed via chronoamperometry at a fixed bias of -3.50 mV over 100 hours.

3. Computational Methods

DFT simulations were conducted using CP2K, employing the Perdew-Burke-Ernzerhof (PBE) exchange-correlation functional. The DZVP-MOLOPT basis set was utilized, with core electrons modelled using the dual-space Goedecker–Teter–Hutter pseudopotentials. The plane-wave cut-off energy was set at 500 Ry, with a relative cut-off of 50 Ry. Convergence for the self-consistent field loops was defined by a total energy change smaller than 10−8 Ry between successive iterations. A Fermi-Dirac smearing at 300 K was used for electronic occupations.

Starting structures were obtained from the Materials Cloud, considering a crystalline $Cu_3Pd_{13}S_7$. Three examples were structured by considering atoms within a spherical cut-off of 0.6-1 nm. Structural relaxations were considered converged when all atomic forces were below 0.5 meV $Å^{-1}$.

Inequivalent adsorption sites were sampled by iteratively positioning an H atom in the vicinity of surface sites; the Python code used to select these positions is available at (*link to be added upon publication*). A total of 103 H adsorption relaxations were then conducted.

The H adsorption energy (H*) is calculated as:

$H^* = E_{H^*} - E_{cluster} - 1/2\ H_2$ (1)

Where E_{H^*}, $E_{cluster}$, 1/2 H_2 respectively label the total energy of the system in the presence of adsorbed H, the bare cluster, and the total energy of H_2 in vacuum. In the analysis, we consider only adsorptions where the E_{H^*} and $E_{cluster}$ structures are not characterised by a too drastic difference in their geometry (e.g., due to a rearrangement), an event which we observe in 10 out of the 103 different relaxations.

All DFT inputs and outputs, and plotting scripts are available via the Materials Cloud repository (*link to be added upon publication*). A cut-off distance of 2.2A from the H atom is adopted to define the adsorption site (top = 1 nearest neighbour atom, bridge = 2 nearest neighbour atoms, hollow = 3 or 4 nearest neighbour atoms). Pd atom neighbours are enumerated by using a 3.2 Å cut-off instead.

4. Additional Characterisation

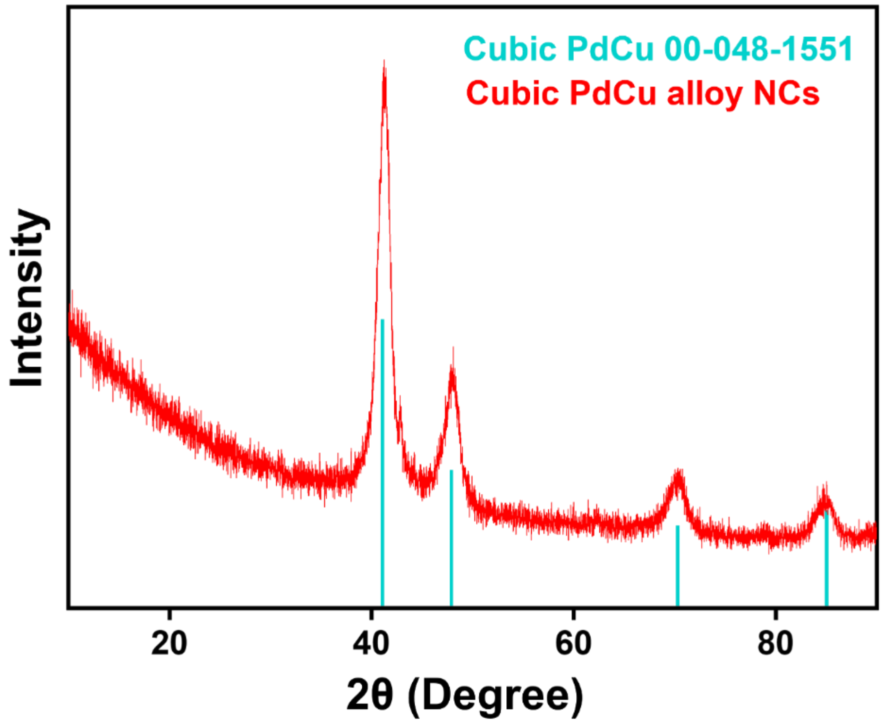

Figure S1. Powder XRD patterns of PdCu alloy NCs.

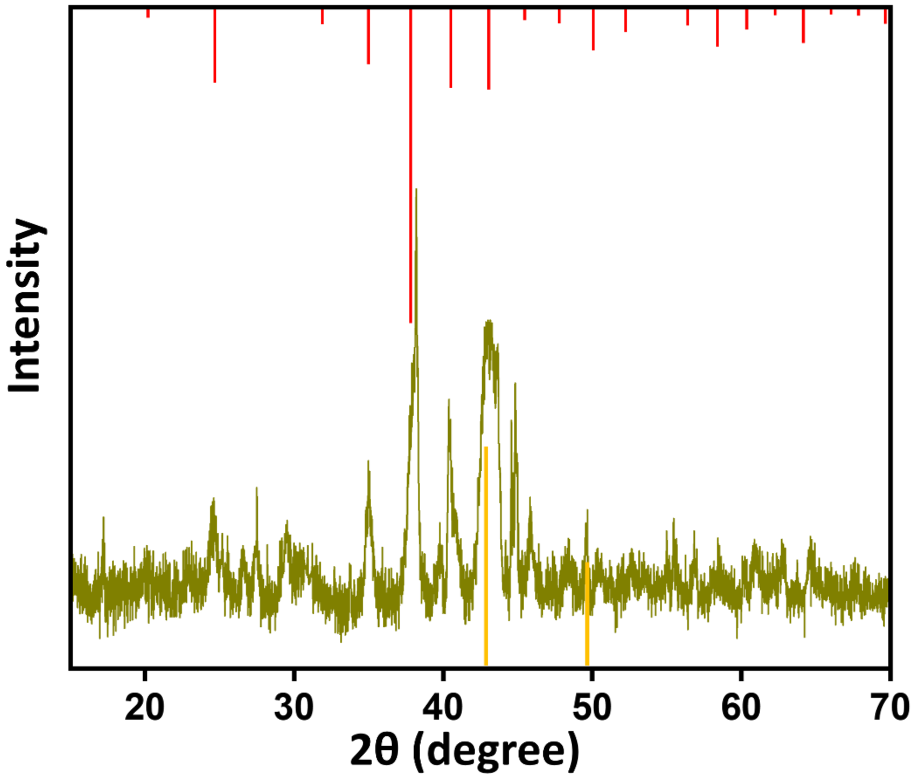

Figure S2. Powder XRD patterns of intermediate samples collected after 1 min of annealing at 180°C.

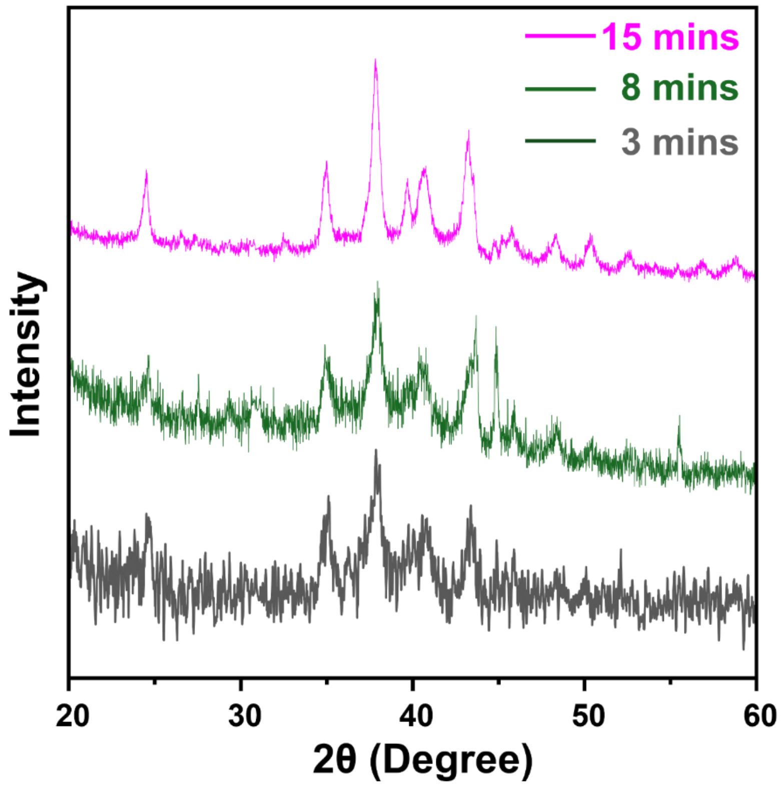

Figure S3. Powder XRD patterns of aliquots collected at different time intervals during the NHCs at 180°C.

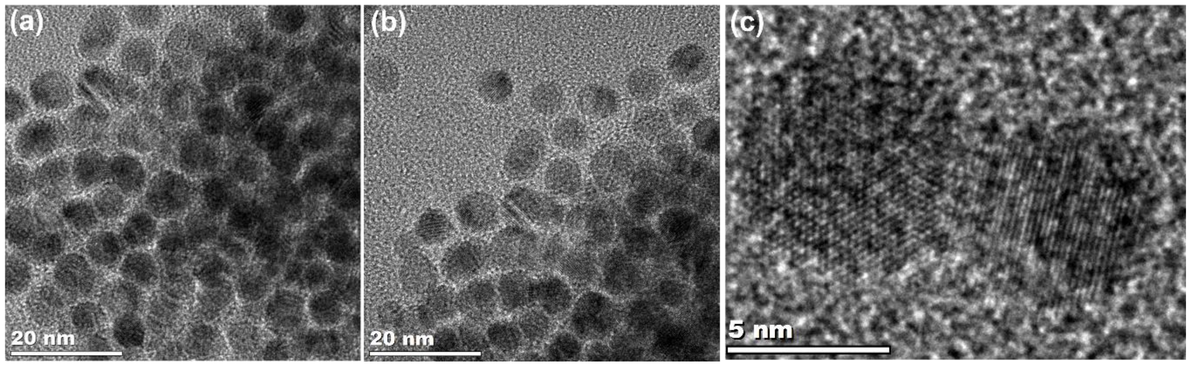

Figure S4. Low magnification and HRTEM image of PdCu alloy

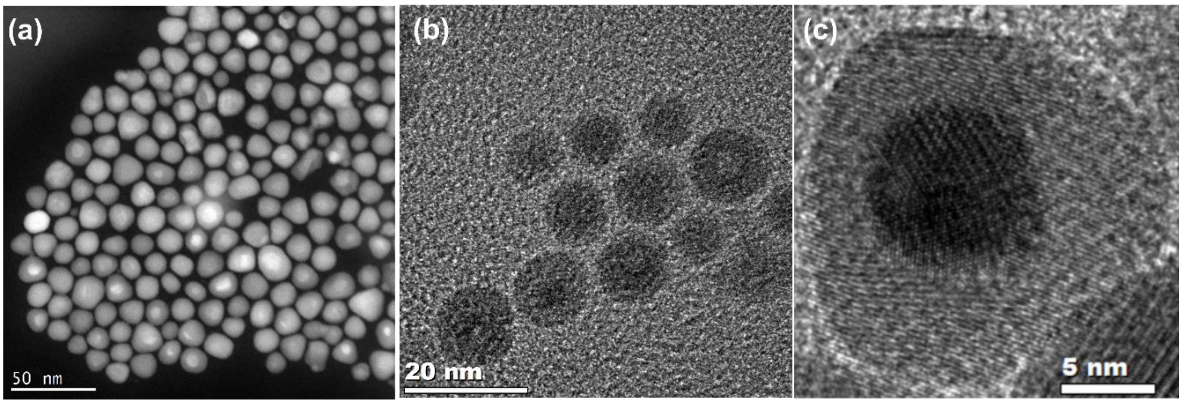

Figure S5. Additional STEM and HRTEM images of Pd-$Cu_3Pd_{13}S_{6.65}Te_{0.35}$ NHCs

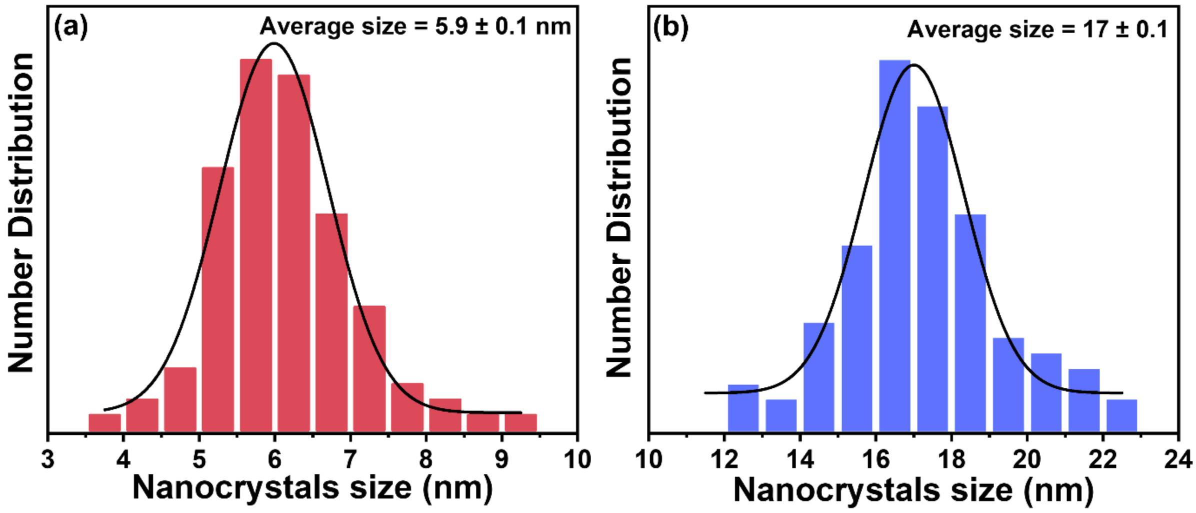

Figure S6. Size distribution analysis for (a) PdCu alloy and (b) Pd-$Cu_3Pd_{13}S_{6.65}Te_{0.35}$ NHCs (sample size 100 particles).

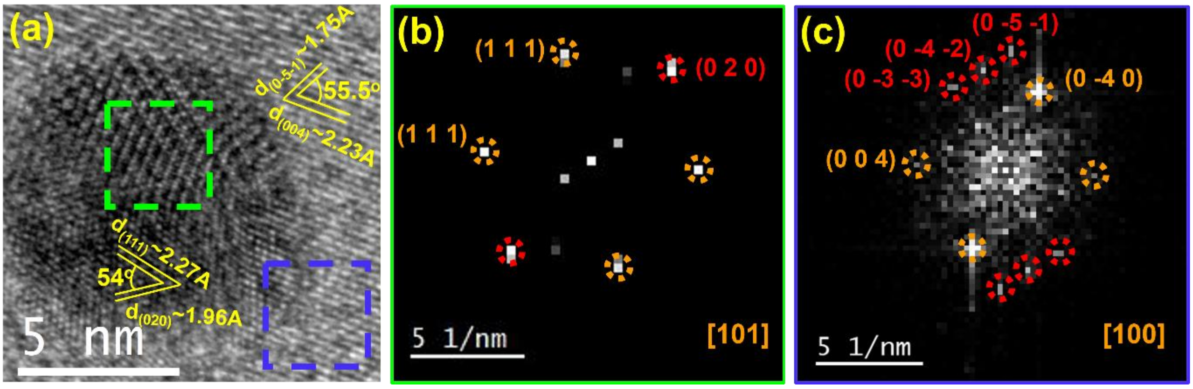

Figure S7. Additional HRTEM analysis image of the NHC (a) and their corresponding FFT (b and c).

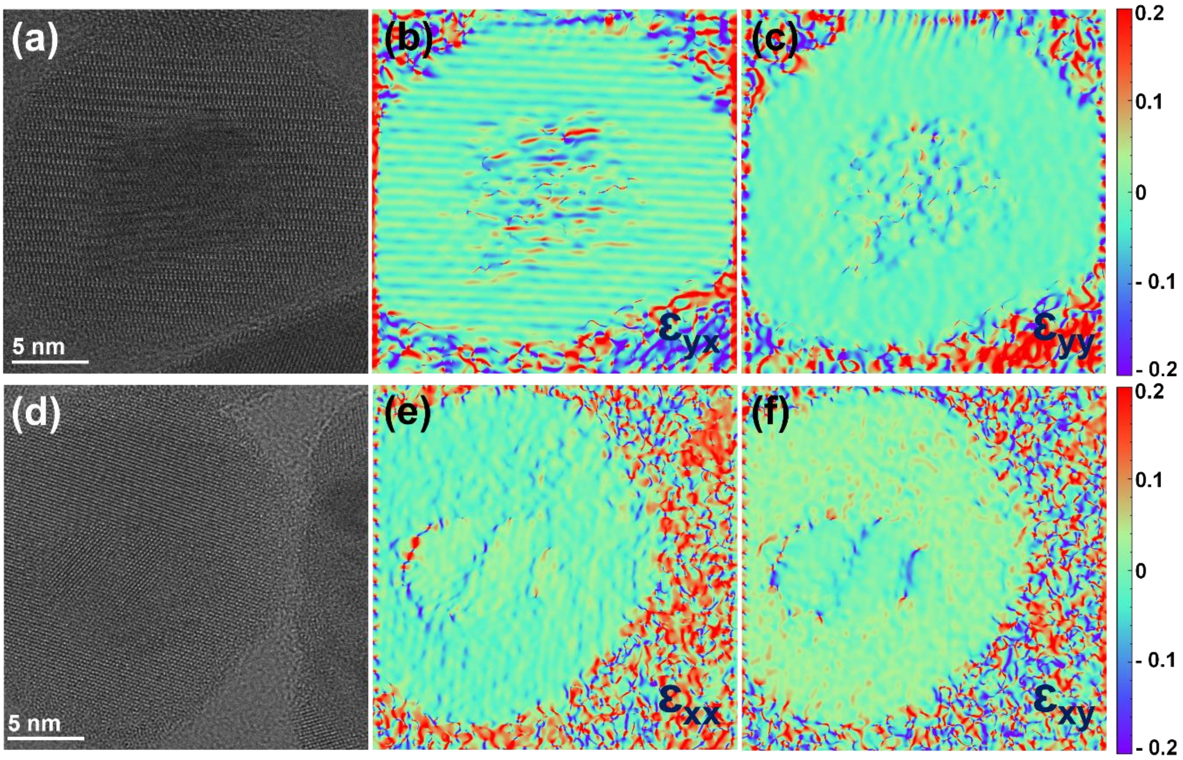

Figure S8. Additional HRTEM images (a and d) and their respective *strain maps* (b-c) ε_{yx}, ε_{yy} *and (e-f)* ε_{xx}, ε_{xy} *(shear) strain show the presence of strain at the interface.*

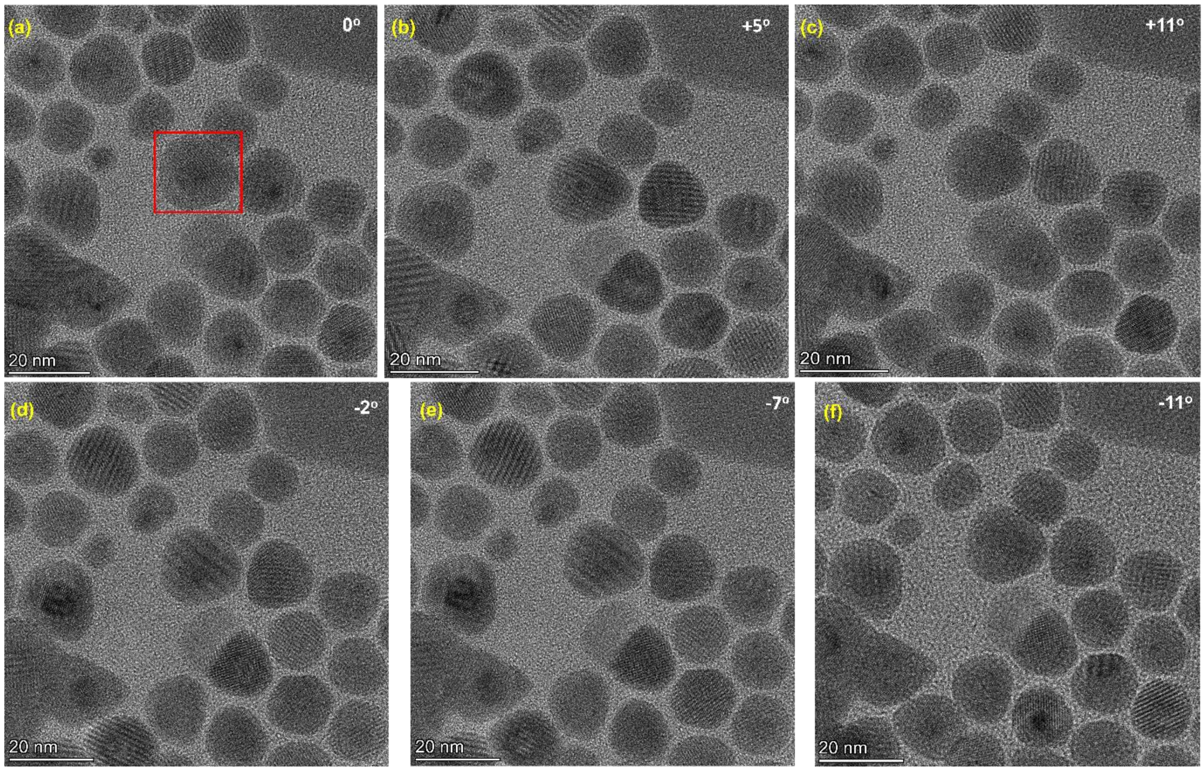

Figure S9. Low resolution bright field TEM of complete tilt series from -11° to +11°

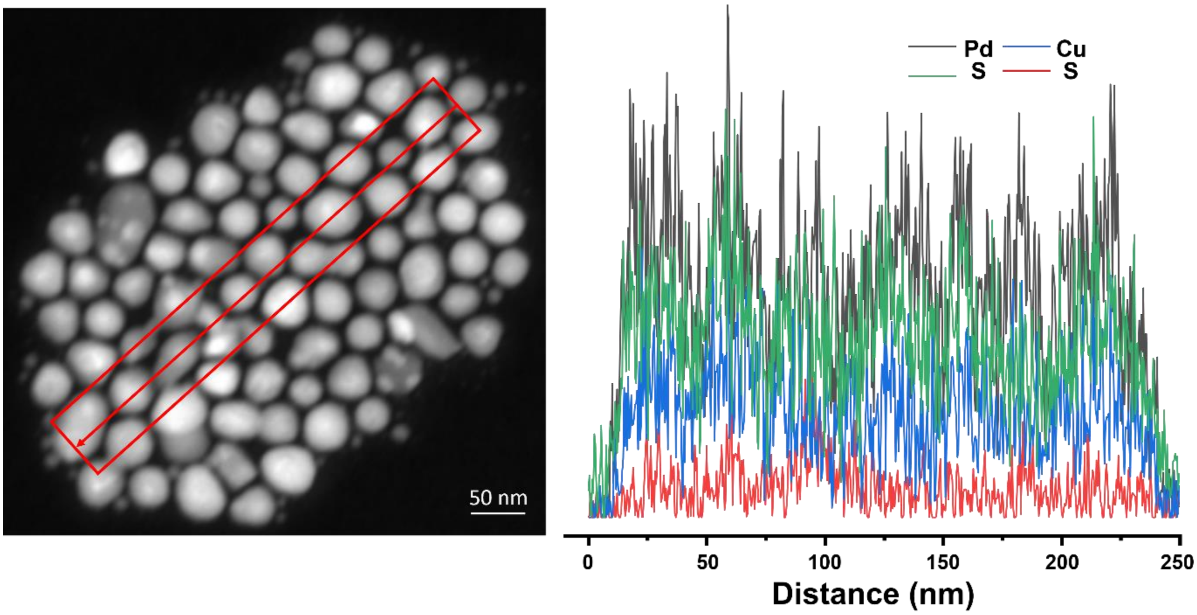

Figure S10. STEM-EDS line scans of aliquots collected after 15 mins annealing at 180°C.

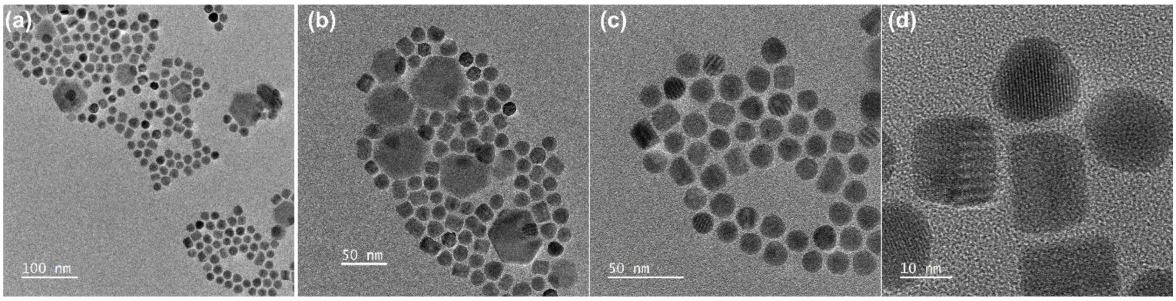

Figure S11. TEM images of cross-nucleated NCs formed at 250°C.

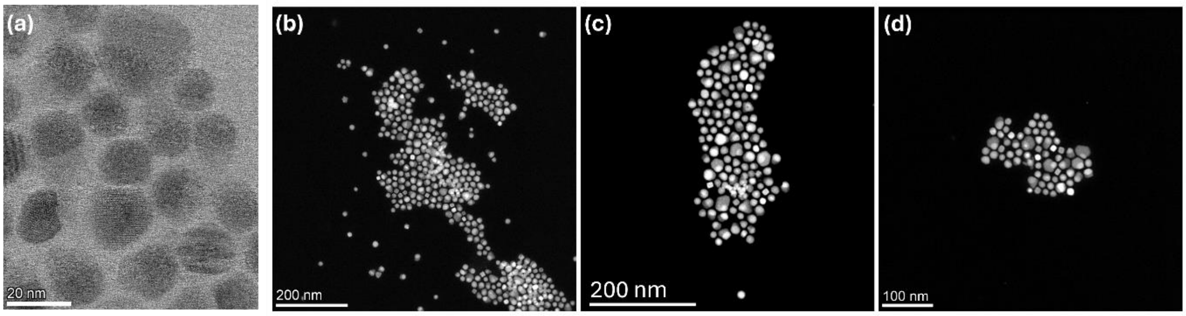

Figure S12. TEM and HAADF-STEM images of intermediates confirming Janus-like structures.

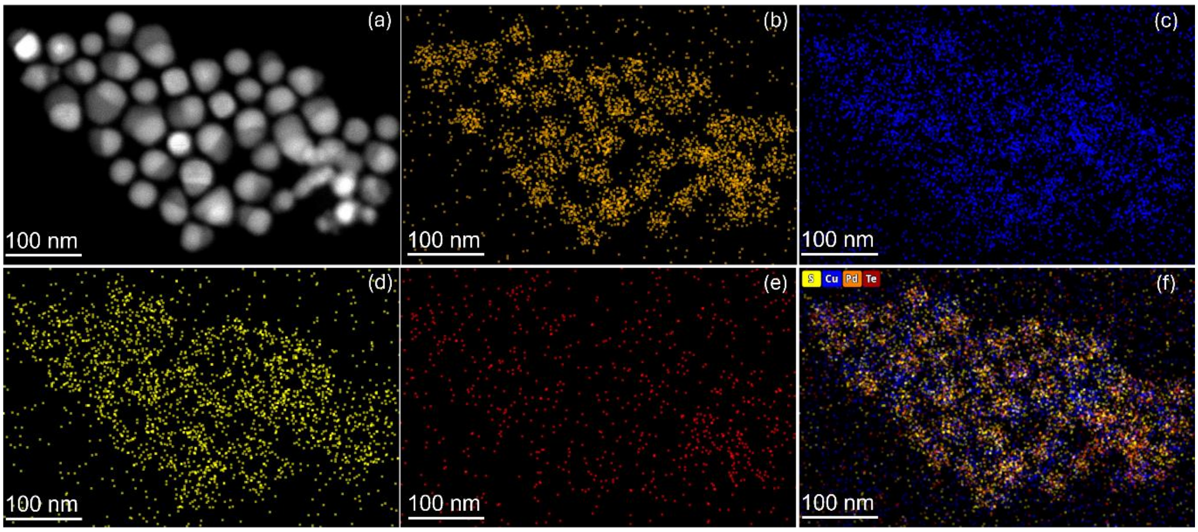

Figure S13. HAADF-STEM EDS mapping of intermediates collected after 1 min of reaction. 180°C.

Table S1. ICP-OES analysis of the NHCs.

Element	Measured Concentration (wt %)	Percentage (%)
Pd (10^{-5} M)	**74.30**	**50**
Cu (10^{-5} M)	**7.92**	**12.5**
S (10^{-5} M)	**14.10**	**35**
Te (10^{-5} M)	**1.65**	**2.5**

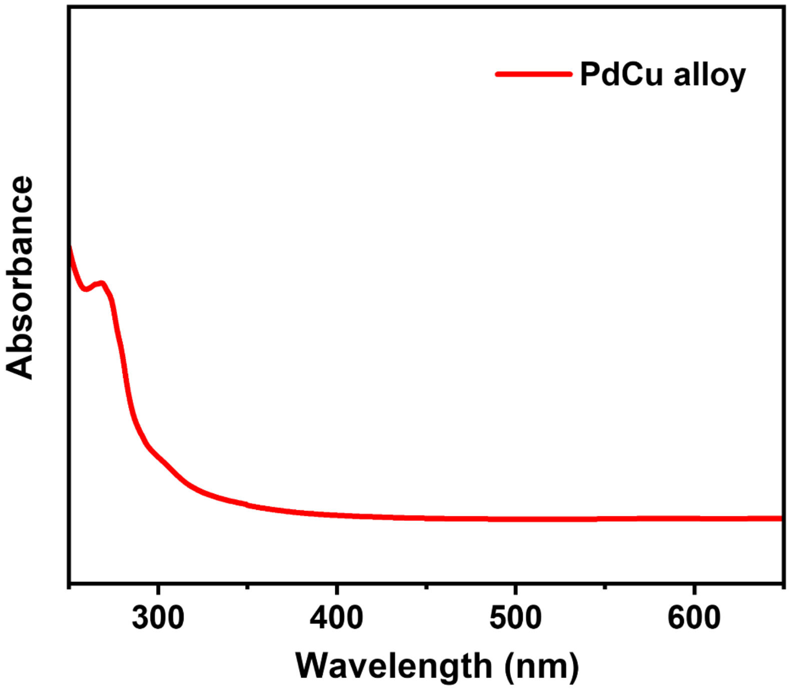

Figure S14. UV-Vis spectra of PdCu alloy.

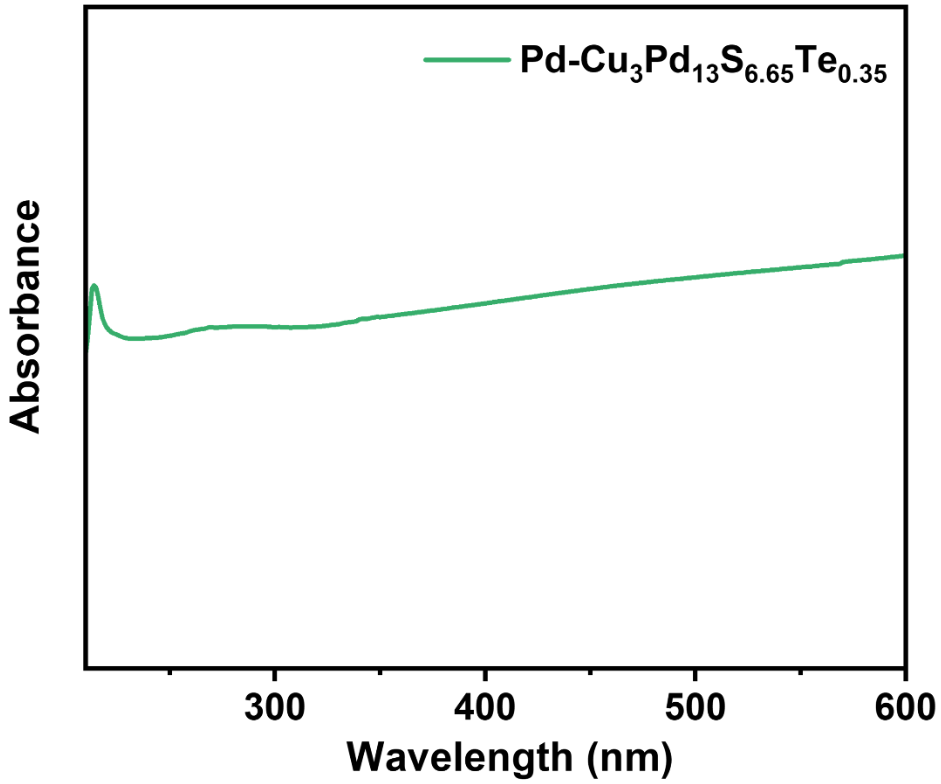

Figure S15. UV-Vis spectra of Pd-$Cu_3Pd_{13}S_{6.65}Te_{0.35}$

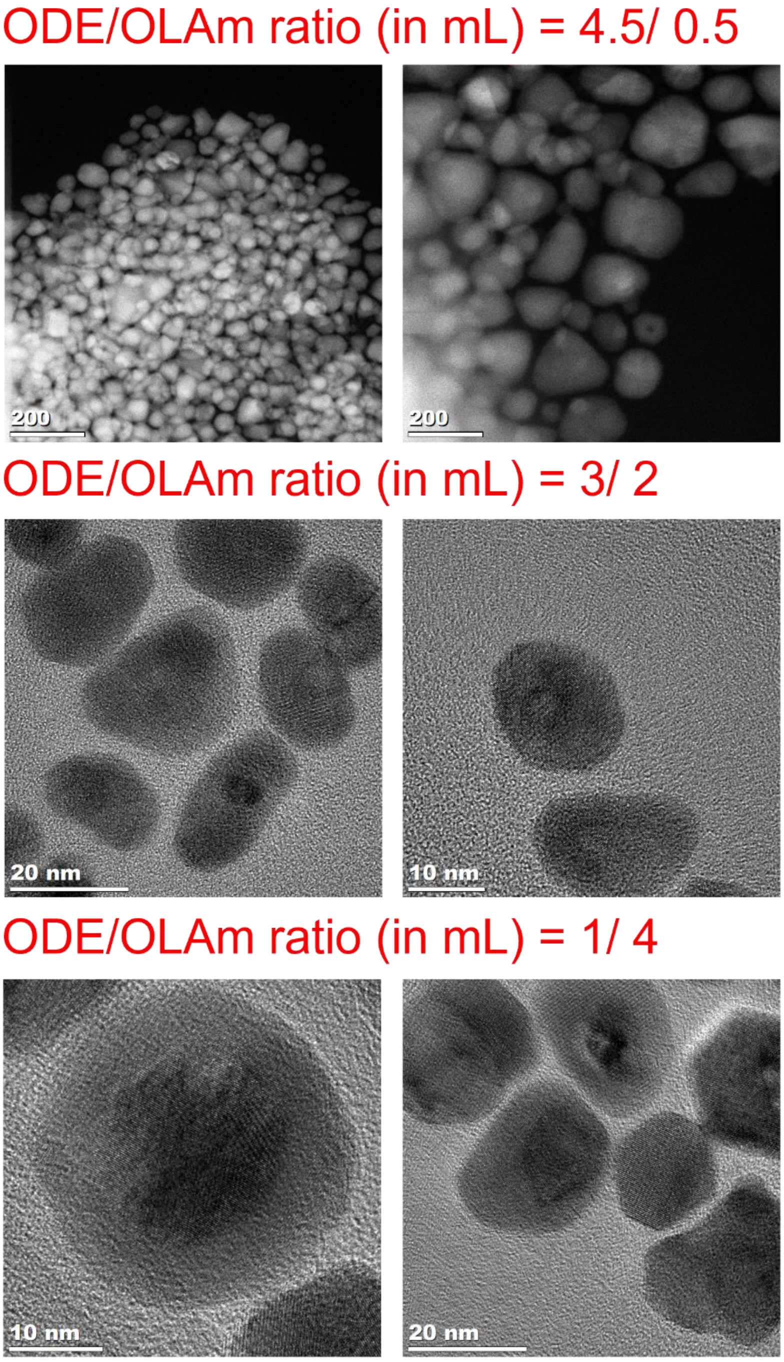

Figure S16. TEM images of NHCs formed when the ODE/OLAm ratio was varied.

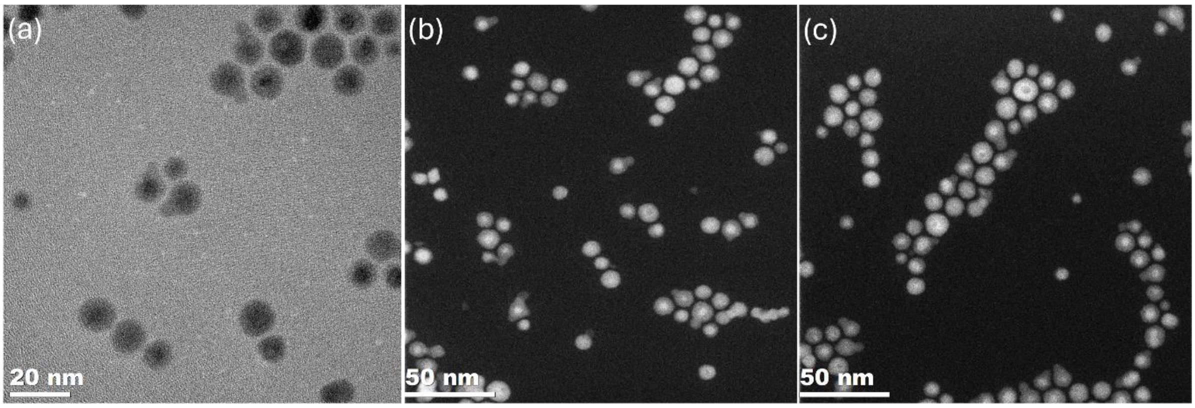

Figure S17. TEM and HAADF STEM images of tadpole-like NHCs formed when elemental S precursor was increased in the reaction.

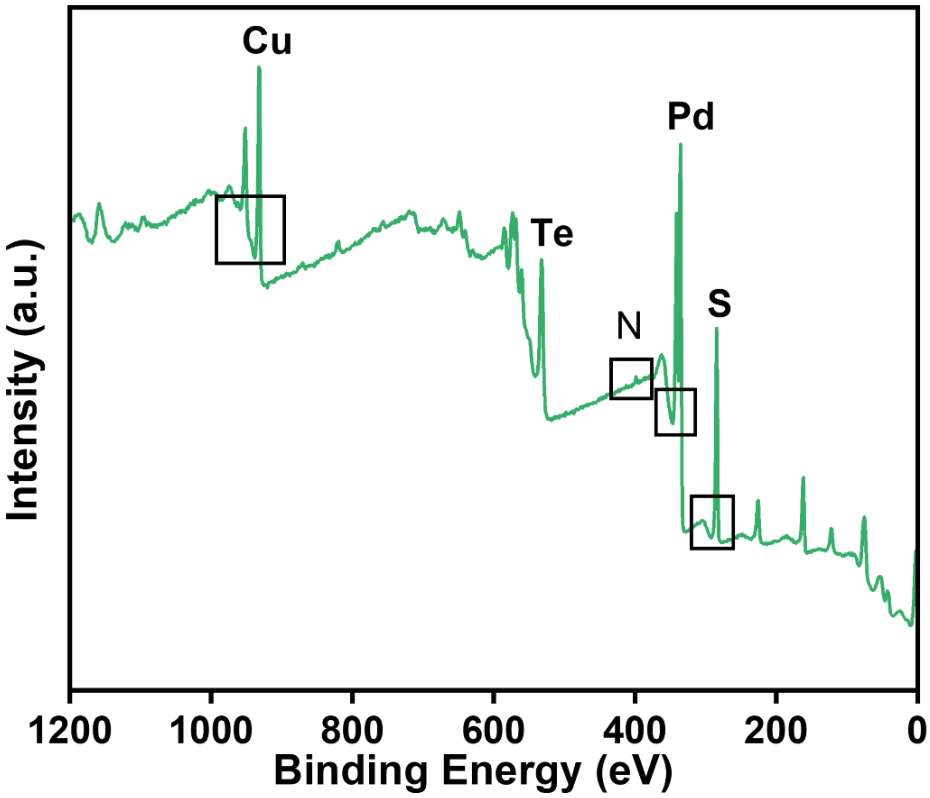

Figure S18. Full X-ray photoelectron spectroscopy (XPS) spectra for Pd-$Cu_3Pd_{13}S_{6.65}Te_{0.35}$

Table S2. Reported metal chalcogenide and metal-metal chalcogenide heterostructure-based HER electrocatalysts.

Sl. No.	Catalyst	Overpotential (mV)	Current Density (mA/cm^2)	Reference
1	Pd-$Cu_3Pd_{13}S_{6.65}Te_{0.35}$	107	10	This report
2	PdCu@Pd NCs	250	10	1
3	$Pd_{16}S_7$/CNFs	245	10	2
4	$Pd_{16}S_7$/MoS_2/CNFs	83	10	2
5	MoS_2/CNFs	183	10	2
6	ReS_2/Ni_3S_2	106	10	3
7	V-Ni_3Se_2	275	500	4
8	Au-$Cu_{2-x}Te$	282	10	5
9	$Cu_{2-x}Te$/hyd	347	10	6
10	$NiTe_2$	125 ± 10	10	7
11	Ni/NiS/P,N,S-rGO	155	10	8
12	$FeSb_2$	54.9	10	9
13	MoP	90	10	10

Table S3. Interpretation of electrochemical parameters derived from Nyquist plots using an electrochemical equivalent circuit model.

Sample	R_s (Ω)	R_{ct} (Ω)
Pd-$Cu_3Pd_{13}S_{6.65}Te_{0.35}$	4	32
CP	2	336

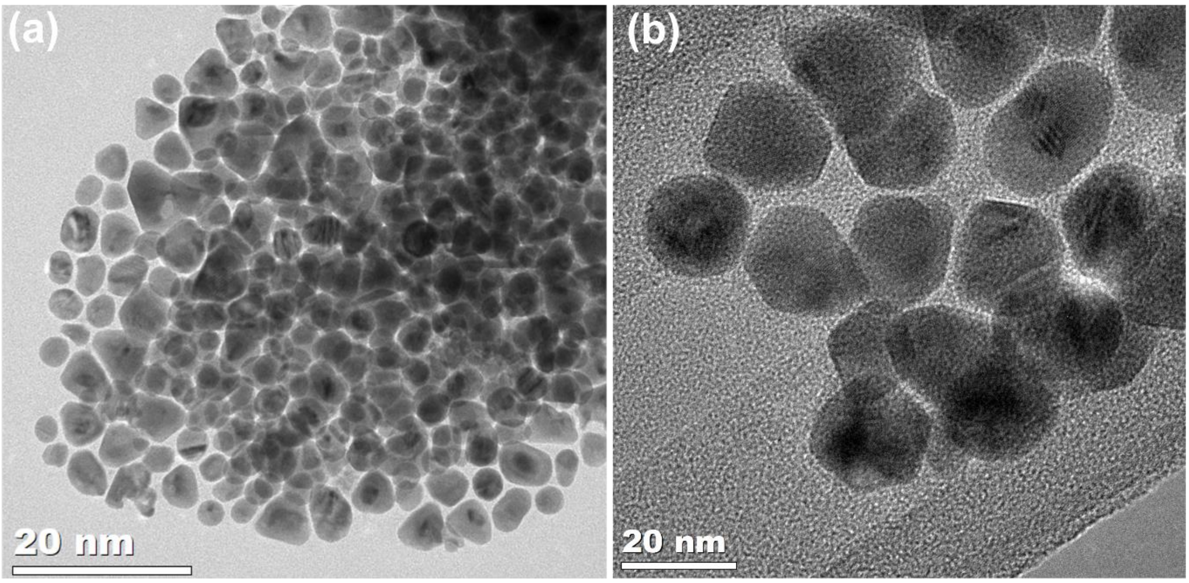

Figure S19. TEM images of post-catalytic samples.

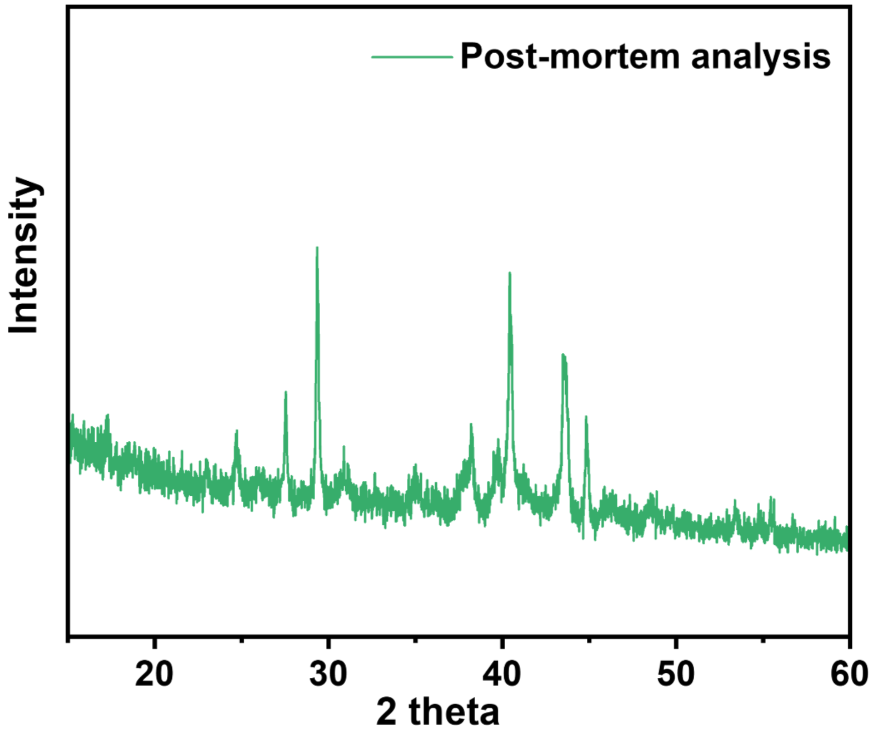

Figure S20. Post-catalytic XRD measurement patterns for Pd-$Cu_3Pd_{13}S_{6.65}Te_{0.35}$ NHCs.

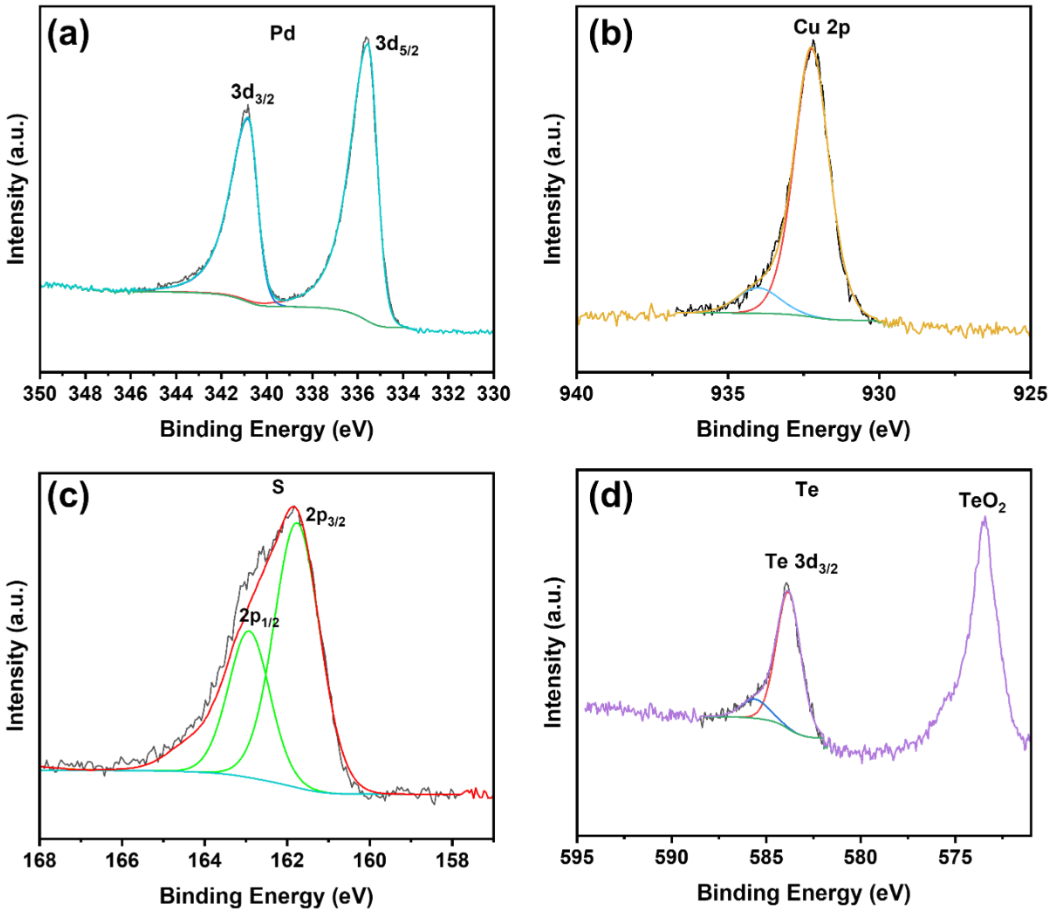

Figure S21. Post-catalytic XPS analysis spectra of Pd-$Cu_3Pd_{13}S_{6.65}Te_{0.35}$ NHCs

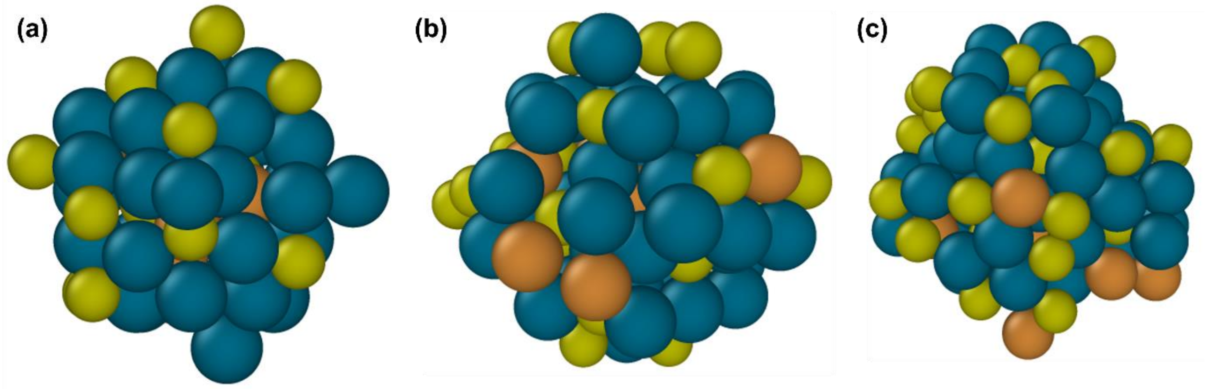

Figure S22. Structures used to sample possible non-equivalent adsorption sites present in a $Cu_3Pd_{13}S_7$.

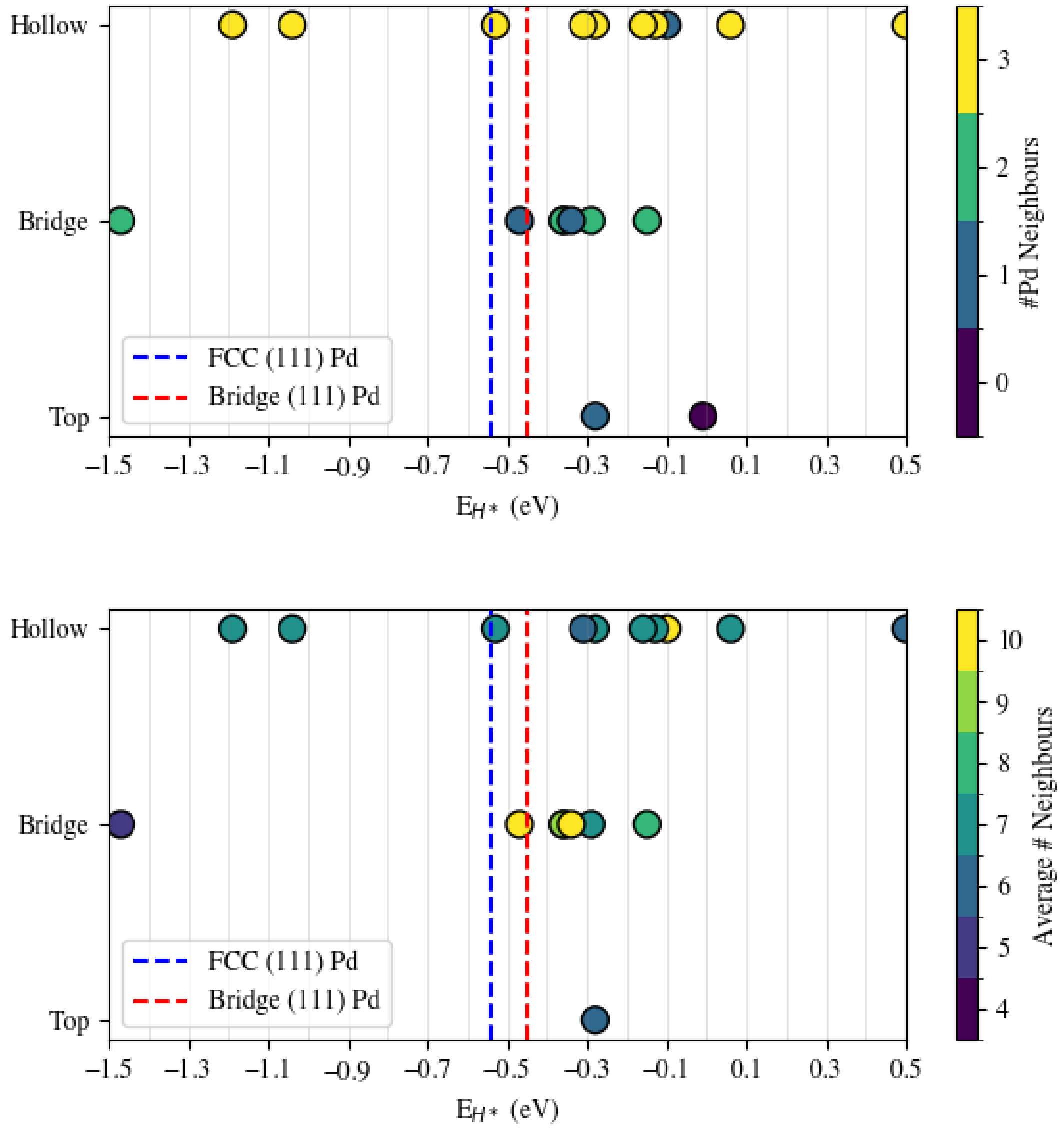

Figure S23. Map of H* adsorption energies as a function of the adsorption mode (Top, Bridge, Hollow – defined as a function of the number of atoms within 2.1Å from the adsorbed H (Top=1, Bridge=2, Hollow=>3) in the $Cu_4Pd_{36}S_{17}$ system. In the top panel, the color bar reports the number of Pd atoms appearing in the adsorption site, in the lower panel, the color bar reports their average coordination (if present). No adsorption site can be mapped (within this local representation) to reference ones in a (111) Pd surface. H* adsorption energies corresponding to high activity are nevertheless observed.

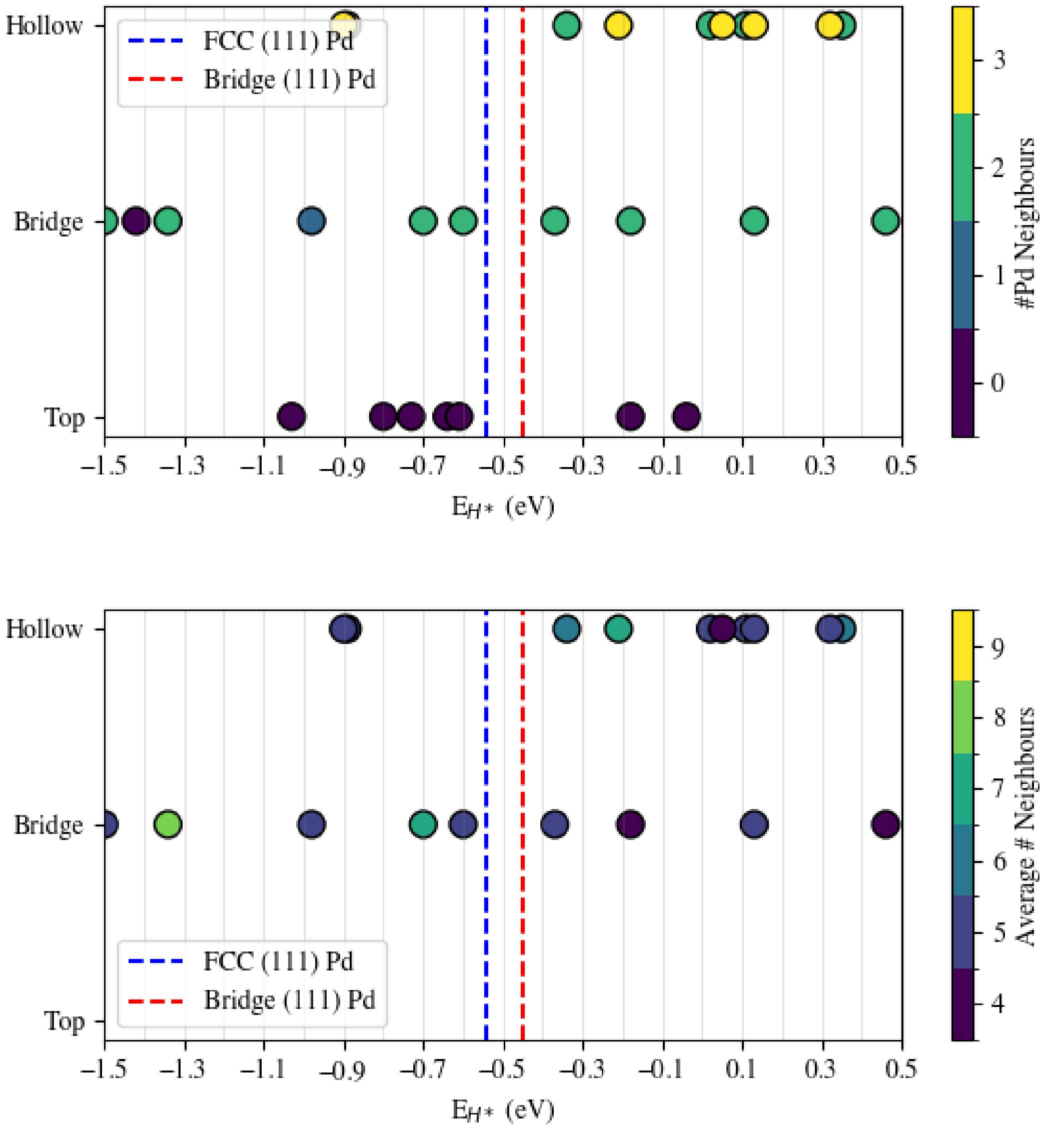

Figure S24. Map of H* adsorption energies as a function of the adsorption mode (Top, Bridge, Hollow – defined as a function of the number of atoms within 2.1Å from the adsorbed H (Top=1, Bridge=2, Hollow=>3) in the $Cu_8Pd_{54}S_{29}$ system. In the top panel, the color bar reports the number of Pd atoms appearing in the adsorption site; in the lower panel, the color bar reports the average coordination of Pd atoms (if present). No adsorption site can be mapped (within this local representation) to reference ones in a (111) Pd surface. H* adsorption energies corresponding to high activity are nevertheless observed.

5. References

1. Li, J.; Li, F.; Guo, S-X.; Zhang, J.; Ma, J. PdCu@Pd Nanocube with Pt-like Activity for Hydrogen Evolution Reaction. *ACS Appl. Mater. Interfaces* **2017**, 9, 8151–8160

2. Wen, Y.; Zhu, H.; Zhang, L.; Hao, J.; Wang, C.; Zhang, S.; Lu, S.; Zhang, M.; Du, M. Beyond Colloidal Synthesis: Nanofiber Reactor to Design Self-Supported Core–Shell $Pd_{16}S_7/MoS_2$/CNFs Electrode for Efficient and Durable Hydrogen Evolution Catalysis. *ACS Appl. Energy Mater.* **2019**, 2, 2013–2021

3. Lu, X.; Liu, R.; Wang, Q.; Xu, C. In Situ Integration of ReS_2/Ni_3S_2 p-n Heterostructure for Enhanced Photoelectrocatalytic Performance. *ACS Appl. Mater. Interfaces* **2019**, 11, 40014–40021

4. He, D.; Cao, L.; Huang, J.; Feng, Y.; Li, G.; Yang, D.; Huang, Q.; Feng, L. Rational Design of Vanadium-Modulated Ni_3Se_2 Nanorod@Nanosheet Arrays as a Bifunctional Electrocatalyst for Overall Water Splitting, *ACS Sustain Chem. Eng*., **2021**, 9, 12005-12016

5. Sen, S.; Shyamal, S.; Mehetor, S.K.; Sahu, P.; Pradhan, N. Au-Cu_{2-x}Te Plasmonic Heteronanostructure Photoelectrocatalysts. *Phys. Chem. Lett.* **2021**, 12, 11585–11590

6. Kumaravel, S.; Karthick, K.; Thiruvengetam, P.; Merlin, Johny, J.M.; Sankar, S.S.; Kundu, S. Tuning Cu Overvoltage for a Copper–Telluride System in Electrocatalytic Water Reduction and Feasible Feedstock Conversion: A New Approach. *Inorg. Chem.* **2020**, 59, 11129–11141

7. Anantharaj, S.; Karthick, K.; Kundu, S. $NiTe_2$ Nanowire Outperforms Pt/C in High-Rate Hydrogen Evolution at Extreme pH Conditions. *Inorg. Chem.* 2018, 57, 3082–3096

8. Hegazy, M.B.Z.; Berber, M.R.; Yamauchi, Y.; Pakdel, A.; Cao, R.; Apfel, U-.P. Synergistic Electrocatalytic Hydrogen Evolution in Ni/NiS Nanoparticles Wrapped in Multi-Heteroatom-Doped Reduced Graphene Oxide Nanosheets. *ACS Appl. Mater. Interfaces* **2021**, 13, 34043–34052

9. Gujjar, D.; Gujjar, S.; Kandpal, C. Intermetallic $FeSb_2$ in a Multifunctional Role of Highly Selective and Efficient Adsorbent, Catalyst, and HER Electrocatalyst. *ACS Appl. Eng. Mater.* **2023**, 1, 2626-2634

10. McEnaney, J. M.; Crompton, J. C.; Callejas, J.F.; Popczun, E. J; Biacchi, A. J.; Lewis, N. S.; Schaak, R. E. Amorphous Molybdenum Phosphide Nanoparticles for Electrocatalytic Hydrogen Evolution. *Chem. Mater.* **2014**, 26, 4826–4831
